# Exit and Entry Screening Practices for Infectious Diseases among Travelers at Points of Entry: Looking for Evidence on Public Health Impact

**DOI:** 10.3390/ijerph16234638

**Published:** 2019-11-21

**Authors:** Varvara A. Mouchtouri, Eleni P. Christoforidou, Maria an der Heiden, Cinthia Menel Lemos, Margherita Fanos, Ute Rexroth, Ulrike Grote, Evelien Belfroid, Corien Swaan, Christos Hadjichristodoulou

**Affiliations:** 1 Department of Hygiene and Epidemiology, Faculty of Medicine, University of Thessaly, 41222 Larissa, Greece; mouchtourib@med.uth.gr (V.A.M.); elchristof@med.uth.gr (E.P.C.); 2 Robert Koch Institute, Department for Infectious Disease Epidemiology, 13353 Berlin, Germany; anderheidenma@rki.de (M.a.d.H.); rexrothu@rki.de (U.R.); groteu@rki.de (U.G.); 3 Consumers, Health, Agriculture and Food Executive Agency (Chafea), L-2920 Luxemburg, Luxemburg; cinthia.menel-lemos@ec.europa.eu; 4 European Commission, DG Health and Food Safety (SANTE), Unit C3—Crisis Management and Preparedness in Health, Luxemburg, L-2920, Luxembourg; margherita.fanos@ec.europa.eu; 5 National Institute for Public Health and the Environment, RIVM, 3720 BA Bilthoven, The Netherlands; evelien.belfroid@rivm.nl (E.B.); corien.swaan@rivm.nl (C.S.)

**Keywords:** border, screening, points of entry, port, airport, ground crossing, health measure, PHEIC, IHR

## Abstract

A scoping search and a systematic literature review were conducted to give an insight on entry and exit screening referring to travelers at points of entry, by analyzing published evidence on practices, guidelines, and experiences in the past 15 years worldwide. Grey literature, PubMed. and Scopus were searched using specific terms. Most of the available data identified through the systematic literature review concerned entry screening measures at airports. Little evidence is available about entry and exit screening measure implementation and effectiveness at ports and ground crossings. Exit screening was part of the World Health Organisation’s (WHO) temporary recommendations for implementation in certain points of entry, for specific time periods. Exit screening measures for Ebola Virus Disease (EVD) in the three most affected West African countries did not identify any cases and showed zero sensitivity and very low specificity. The percentages of confirmed cases identified out of the total numbers of travelers that passed through entry screening measures in various countries worldwide for Influenza Pandemic (H1N1) and EVD in West Africa were zero or extremely low. Entry screening measures for Severe Acute Respiratory Syndrome (SARS) did not detect any confirmed SARS cases in Australia, Canada, and Singapore. Despite the ineffectiveness of entry and exit screening measures, authors reported several important concomitant positive effects that their impact is difficult to assess, including discouraging travel of ill persons, raising awareness, and educating the traveling public and maintaining operation of flights from/to the affected areas. Exit screening measures in affected areas are important and should be applied jointly with other measures including information strategies, epidemiological investigation, contact tracing, vaccination, and quarantine to achieve a comprehensive outbreak management response. Based on review results, an algorithm about decision-making for entry/exit screening was developed.

## 1. Introduction

Public health events can cause serious crises and damage to the human population if effective frameworks and systems are not in place to prevent, early detect, and respond in a timely manner to health threats. In recent years, events such as the Public Health Emergencies of International Concern of Zika Virus Disease declared in 2016 and Ebola Virus Disease (EVD) outbreak declared in 2014 affected or have/had the potential to seriously affect a large amount of the population. High mobility of populations across borders of countries can contribute to the rapid spread of diseases. Screening measures on travelers at points of entry including airports, ports, and ground crossings can be implemented to prevent international transmission of disease by detecting and prohibiting travel to exposed or ill travelers from affected areas [1,2]. 

The International Health Regulations (IHR) 2005 states in articles 5, 13, 18, 19, and Annex 1B that World Health Organisation (WHO) recommendations in response to a Public Health Emergency of International Concern (PHEIC) may include screening measures at points of entry. Moreover, WHO State Parties must have the capacities to apply entry or exit controls for arriving and departing travelers [3]. Decision 1082/2013/European Union (EU) of the European Parliament and of the Council on serious cross-border threats to health requires that “*Member States and the European Commission shall consult each other within the Health Security Committee (HSC)…That consultation shall be aimed at “…supporting the implementation of core capacity requirements for surveillance and response as referred to in Articles 5 and 13 of the IHR*” [4], including capacities in implementing screening measures at borders. 

Since the entry of IHR 2005 into force, the WHO Director General has declared five PHEIC according to Article 12: in 2009 the Influenza Pandemic (H1N1), in 2014 the Poliovirus situation worldwide, in 2014 and 2019 the Ebola virus disease (EVD) outbreak in West Africa and the Democratic Republic of the Congo respectively, and in 2016 the Zika virus disease [5]. Moreover, in 2003 the Severe Acute Respiratory Syndrome (SARS) outbreak alerted the global community. Temporary recommendations about exit screening in affected countries have been issued by WHO and applied in those countries. Other countries have implemented entry screening measures on travelers arriving from affected countries at ports, airports, and ground crossings, in response to PHEIC or as part of the routine measures to prevent disease introduction to their country [6,7,8,9,10,11]. 

As has been described in WHO technical guidance during past public health events, entry or exit screening measures are generally conducted as a two-step process: primary screening and secondary screening [1,2]. With the primary screening, an initial assessment is carried out by personnel, who may not necessarily have public health or medical training. Activities include visual observation of travelers for signs of the infectious disease, measurement of travelers’ body temperature, and completion of a questionnaire by travelers asking for presence of symptoms and/or exposure to the infectious agent. Travelers who have signs or symptoms of the infectious disease, or have been potentially exposed to the infectious agent, are referred to secondary screening. Secondary screening should be carried out by personnel with public health or medical training. It includes an in-depth interview, a focused medical and laboratory examination and second temperature measurement [1,12]. Specific objectives of entry or exit screening measures can include: identification of ill travelers who may have signs and symptoms, and of travelers who may have been exposed to a hazard and their close contacts; identification of appropriate public health measures, such as treatment, isolation, quarantine and travel restrictions that are commensurate with the risks and do not unduly interfere with international travel; proper collection of information and reporting of public health risks; provision of information and education to the traveling public about health risks. 

Screening measures can be implemented for long-time periods for specific diseases as part of the country’s routine measures to prevent introduction of diseases to the country, or ad hoc after emergent public health events. Moreover, screening measures could be implemented massively to all inbound or outbound travelers at a point of entry, or targeted to specific travel routes (e.g., departing from an affected area) or to specific travelers (e.g., who have been in an affected area). 

Previous attempts to assess the effectiveness of entry and exit screening measures have demonstrated either limited public health impact of such measures [9,13,14], or evidence of success [15] and benefits [6,16]. In the recent published literature, there is a lack of a systematic approach in appraising the evidence for usefulness of screening measures that could help countries in their decision-making on implementing health measures and allocating resources. 

This paper describes the results of a scoping search and of a systematic bibliographic review aiming at giving insight on entry and exit screening referring to travelers at points of entry worldwide, with an emphasis among EU/European Economic Area (EEA) Member States (MS). Evidence from this study was used to inform EU MS in the framework of a training course conducted in 2019 about entry/exit screening structures and processes currently in place in EU MS and worldwide, as well as the strengths, limitations and lessons learnt from applying entry/exit screening at points of entry. Decision-making issues on implementing health measures that are commensurate with the risk, avoiding unnecessary interference with international traffic and trade, and considering business continuity plans are also discussed. Finally, the review explores preparedness issues and the capacities that must be in place at points of entry to implement entry/exit screening for infectious diseases. 

## 2. Materials and Methods

### 2.1. Methods for Scoping Search

#### 2.1.1. Research Question

The scoping search was conducted to answer the following research questions: 

(1) *“What are the practices, guidelines and experiences worldwide on entry and exit screening for infectious diseases to travelers at points of entry that have been published in the past 15 years?”*

(2) *“What are the effects, the benefits and the limitations of entry and exit screening measures for infectious diseases to travelers at points of entry that have been published in the past 15 years?”*

#### 2.1.2. Search Strategy

Grey literature, PubMed and Scopus were searched for relevant documents published in the past 15 years using the following search terms: (exit screening OR entry screening OR border measure) AND (patient OR ill OR sick OR infected OR affected OR exposed OR symptomatic) AND (human OR passenger OR traveler OR traveler OR crew) AND (airport OR aerodrome OR airdrome OR seaport OR port OR point of entry OR port of entry). 

The scoping search was conducted independently of the systematic bibliographic review, which has been presented in the last paragraph of the first chapter. 

#### 2.1.3. Inclusion and Exclusion Criteria

Inclusion criteria were: articles or reports or other documents published in peer-reviewed journals or national and international organizations’ publications, from 2003 until May 2018 that report practices, implementation of guidelines, experiences, structures, processes, evaluation results about national routine or ad hoc entry or exit screening activities referring to travelers at ports or airports or ground crossings, worldwide, performed during serious cross-border health events.

Articles that refer to (a) migrants, refugees, and asylum seekers were excluded, except when related to response to a global health emergency, (b) screening of diseases that were not part of a global health emergency response, (c) entry or exit screening measures that were part of response to a specific outbreak on board an airplane or a ship and not part of a country response to a global health threat.

### 2.2. Methods for Systematic Bibliographic Review

#### 2.2.1. Research Questions

The bibliographic review was conducted to answer the following research questions: 

(1) What are the public health impact and the cost-effectiveness of implementing entry or exit screening among travelers for infectious diseases at ports, airports, and ground crossings by using different assessment methods? 

(2) What are the good practices for implementing entry or exit screening among travelers for infectious diseases at ports, airports, and ground crossings? 

#### 2.2.2. Specific Objectives

The specific objectives of the bibliographic review were the following: 

(1) Objective 1

To identify practices and experiences on entry and exit screening referring to travelers worldwide by using the evidence found in the literature and reports published by competent authorities.

(2) Objective 2

To identify the lessons learnt from entry and exit screening referring to travelers at points of entry worldwide by using the evidence found in the literature and reports published by competent authorities or international organizations. 

(3) Objective 3 

To critically appraise the evidence for the public health impact and/or cost-effectiveness of entry and exit screening measures implemented on a routine basis or ad hoc basis to travelers worldwide. 

#### 2.2.3. Search Strategy

(1) Search topic and concepts

The research topic concerns the public health impact, the cost-effectiveness and the practices and experiences for implementing entry or exit screening among travelers for infectious diseases at ports, airports, and ground crossings, by using different assessment methods. 

The search concepts used for the above-mentioned topic are: (a) **public health event**: infectious diseases in humans, (b) **type of measure**: entry screening or exit screening, (c) **population of interest**: travelers (crew and passengers) crossing borders, (d) **setting**: points of entry: airport, port, ground crossing, (e) **outcome**s: cost-effectiveness, public health impact. 

For the purposes of this bibliographic review, the following definitions have been used for “entry screening” and “exit screening” terms: 

(a). “Entry screening” are the public health measures implemented at points of entry (ports, airports, ground crossings) on travelers (crew and passengers) arriving to a country, with the purpose to assess the exposure to a biological agent (bacterium, virus, parasite) and/or the presence of symptoms. Entry screening is part of the international and domestic policies of competent authorities to control disease spread and to minimize impact on travel and trade, which can be severely affected by absence of adequate measures or lack of capacity to implement these measures [17]. 

(b). “Exit screening” are the public health measures implemented at points of entry (ports, airports, ground crossings) on travelers (crew and passengers) departing from a country, with the purpose to assess the exposure to a biological agent (bacterium, virus, parasite) and/or the presence of symptoms. Exit screening is part of the international and domestic policies of competent authorities to control disease spread and to minimize impact on travel and trade, which can be severely affected by absence of adequate measures or lack of capacity to implement these measures [17].

(2) Search resources and terms

PubMed and Scopus were searched to identify relevant publications in peer-reviewed journals. The search terms used are presented in Table 1.

To identify the relevant grey literature the following websites were searched: WHO (headquarters, regional offices), European Center for Disease Prevention and Control (ECDC), US Centers for Disease Control and Prevention (CDC), Public Health Agencies and Surveillance Centers of EU/EEA MS and non-EU EU/EEA countries and the following organizations: International Civil Aviation Organisation (ICAO), International Air Transport Association (IATA), Collaborative Arrangement for the Prevention and Management of Public Health Events in Civil Aviation (CAPSCA), Airport Council International (ACI), Cruise Lines International Association (CLIA), International Shipping Federation (ISF), International Union of Railways (UIC), Intergovernmental Organisation for International Carriage by Rail (OTIF), Organisation for Cooperation between Railways (OSJD), International Rail Transport Committee (CIT), European Rail Research Advisory Council (ERRAC). A detailed list of all websites searched is included in Appendix A (Table A1). In addition, the reference lists from the relevant articles (hand search) was conducted and the eligible articles that were identified were included in the study.

Moreover, websites of WHO and of national public health institutes were searched in order to identify publicly available surveillance data about the number of cases of the diseases targeted by the screening measures and reported during the period of entry/exit screening measure implementation in the country. These were used to make comparisons with the number of confirmed cases identified through the entry screening measures. 

Additionally, the WHO website was searched to identify the temporary recommendations issued by WHO in response to the four above-mentioned PHEIC and recommended measures issued after other emergent public health events. Data from the WHO relevant reports were extracted about the timeframe of recommended screening measure implementation, the methods for screening, and the areas, as well as the advice for travel restrictions.

Two researchers checked the documents independently (titles, abstracts, full texts) for the eligibility criteria.

#### 2.2.4. Inclusion and Exclusion Criteria

(1) Inclusion and exclusion criteria

The inclusion and exclusion criteria used for the bibliographic review are presented in Table 2. Appendix B presents the questionnaire used to check documents for eligibility criteria. 

#### 2.2.5. Analysis of the Literature

(1) Quality of articles appraisal 

The quality of articles included in the review were assessed based on completing the inclusion criteria. 

(2) Data extraction 

Specific questions were used by the two researchers to systematically extract the data from the articles, as shown in the questionnaire presented in Appendix C. Two researchers extracted independently data from the eligible articles. The following data were extracted from the papers/reports that fulfilled the inclusion/exclusion criteria of the review:

- Type of screening (entry, exit)
- Types of infectious disease or diseases that entry and exit screening was targeting
- Type of points of entry: airports, ports, ground crossings
- Screening carried out on a routine basis or on an ad hoc basis after a public health event has occurred
- Methods used in entry/exit screening (primary, secondary, questionnaire, body temperature, technology used etc.)
- Type of technology used (thermometers, scan cameras etc.)
- After screening, the applied diagnosis protocol (laboratory and clinical examination)
- Number of cases identified and the total numbers of travelers screened
- Percentage of persons positive to screening finally diagnosed
- Percentage of persons diagnosed with a different disease from the initially targeted
- The applied protocol for diagnosis and management of cases
- Health measures applied to the traveler and the environment
- General screening or targeted screening: outbound country, travelers directly arriving from affected countries, nationality, travelers in-transit
- Inter-sectorial collaboration and coordination processes
- Involved officers: public health officers, ministry officers, regional health system, national health system, NGOs, else
- Concrete example of entry/exit screening
- Practices, experiences, and lessons learnt reported
- Challenges reported (limitations, failures, mishaps)
- Bad practices reported
- Methods used to assess the public health impact of the entry/exit screening and their result
- Methods used to appraise the cost-effectiveness of screening method and results
- Evaluation of method results: sensitivity, specificity, false positive/negative (of screening method), positive and negative predictive values
- Decision-making level: public health officers, ministry officers, regional, national, intersectoral collaboration, health, and border authorities
- Communication channels
- Notification practices between neighboring and possibly affected countries
- Specific timeframe referred and duration

#### 2.2.6. Ethical Considerations

This bibliographic review concerns a literature review of already published material, and therefore ethics approval was not required.

## 3. Results

### 3.1. Results of Scoping Search

The scoping search identified 82 scientific articles, six documents/reports from public health agencies of countries and 26 guidelines/reports from international organizations. 

In total, 114 identified documents of scoping research can be categorized into the following categories:

(a) Assessment for imported cases notification of infectious diseases
(b) Dengue entry screening at airports
(c) Preparedness and response planning for Ebola Virus Disease
(d) Entry/exit screening measures for Ebola Virus Disease experience
(e) Studies about evolution and predictions of Ebola Virus Disease spread
(f) Entry/exit screening measures for infectious diseases
(g) Influenza
(h) Pandemic influenza preparedness
(i) International air travel and infectious diseases
(j) Preparedness planning for infectious disease
(k) Screening measures at ground crossing
(l) Sever Acute Respiratory Syndrome
(m) Zika Virus Disease

The list of documents identified through the scoping search can be found in Appendix D, and is presented in thematic sections and in alphabetical order including authors, title, and year of publication. Twenty-four articles in Appendix D were also identified through the systematic bibliographic review search as described in Section 2.2.3. 

Table 3 summarizes the degree of success of the primary objective of screening measures in identifying ill or exposed travelers, the limitations and both the beneficial and adverse concomitant effects of entry and exit screening at points of entry for SARS, Influenza Pandemic (H1N1) 2009 and EVD, as reported by the authors. Results concerning measures implemented as part of long-term measures for Dengue fever and not as a response to emergencies are not presented in Table 3. 

Screening measures as reported in the published literature were decided to be applied at the specific setting and situation, and generalizing conclusions was not considered to be appropriate [14]. Disease virulence, type, and severity of symptoms, length of incubation period, proportion of asymptomatic carriers, transmissibility, period of communicability, and mode of transmission were factors that determined the degree of success of screening measures depending on the disease, as well as the extent and evolution of the outbreak and the phase that measures were applied [18]. Finally, the country characteristics seemed to play a role such as whether the country was an island country or shared borders or had direct connections of flights or ship itineraries with affected countries [19]. For the previously mentioned reasons, no general conclusions about the impact of entry or exit screening for all infectious diseases could be drawn, and appraisal of impact should be done considering each specific disease and the context of screening measure implementation.

Data from the scoping search were used to develop the algorithm for making evidence-based decisions in implementing entry and exit screening measures (Appendix E). 

**Table 3:** Degree of success, limitations and concomitant effects of entry and exit screening at points of entry for Severe Acute Respiratory Syndrome (SARS), Influenza Pandemic (H1N1) 2009 (A(H1N1)pdm09) and Ebola Virus Disease (EVD).

Degree of Success in Identifying Ill or Exposed Travelers	Limitations	Concomitant Effects	
		Beneficial	Adverse
**Influenza A(H1N1)pdm09^˅^ [20,21]** **Sensitivity: 6.67% (95% CI, 1.40%–18.27%)** **Specificity: 99.10% (95% CI, 99.00%–100.00%)** **EVD ^˄^ [22]** **Sensitivity: 0%** **Specificity: 99.79%** **SARS ^˅^ [8,9,13,23]** **Entry screening measures did not detect any confirmed SARS cases in Australia, Canada, and Singapore.**	Influenza A(H1N1)pdm09 ∙ Screening cannot detect incubating or asymptomatic travelers ^˅^ I [24] SARS ∙ False declarations by passengers about exposure and disease signs and symptoms ^˅^ S [8] ∙ Antipyretic drugs can be used by travelers to conceal fever ^˅^ S [8] ∙ Questionnaires asking about exposure and thermal scanning machines, were non-specific for SARS ^˅^ S [9] ∙ The frequency of SARS among international passengers arriving or departing was low resulting in low positive predictive value ^˅^ S [9] ∙ The de facto point of entry into the healthcare system for travelers with serious infectious diseases was found to be the in-country, acute care facilities (hospitals, clinics, and physicians’ offices) and not the airports ^˅^ S [9] ∙ Language barriers—flight announcements about screening measures and requests for declaring exposures were not understood by passengers ^˅^ S [8] ∙ Exit screening measures may have not dissuading ill travelers from attempting to return home ^‡^ S [25]	Influenza A(H1N1)pdm09 and EVD ∙ Obtaining contact information of travelers to be used if needed for contact tracing or public health observation purposes ^˅^ E,I [6,26] EVD ∙ Educating and informing the traveler passing through the screening points about the public health risks and prevention measures ^˅^ E [6] ∙ Linking the traveler with public health authorities for the duration of the incubation period to facilitate health monitoring and prompt referral for care if they became ill ^˅^ E [6] ∙ Facilitating rapid and appropriate clinical care for ill travelers ^‡^ E [6] ∙ Maintaining confidence that air travel is safe ^˅^ E [6] ∙ Enabling humanitarian and public health organizations to sustain travel to affected areas by regular commercial airline flights, maintaining continued flow of passenger traffic and resources needed for the response to the affected region ^˄^ E [16,27,28] ∙ In EVD-affected countries, laid the foundation for future reconstruction efforts related to borders and travel, including IHR core capacities (e.g., regional surveillance systems, cross-border coordination) ^˄^ E [16] SARS ∙ May have helped dissuade ill persons from traveling by air ^‡^ S [25] ∙ Preserving public confidence ˅S [8,9,29], relieving political and social pressure and limiting negative economic consequences from travel and trade restrictions ^˅^ S [8] ∙ Help avoiding major economic, social and international impact which even a single imported SARS case may have ^˅^ S [23]	EVD ∙ May give to the public a false sense of security ^˅^ E [30] ∙ Stigmatization of travelers under public health observation ^˅^ E [31] SARS ∙ High cost of screening measures ^˅^ S [8,9,13] ∙ Investing in screening measures reduces the resources from other effective measures ^˅^ S [9,23]

^˄^: Exit screening, ^‡^: Entry and Exit screening, ^˅^: Entry screening “S”: Severe Acute Respiratory Syndrome (SARS),”I”: 2009 Influenza Pandemic (H1N1) (influenza A(H1N1)pdm09), “E”: Ebola Virus Disease (EVD).

### 3.2. Results of Systematic Bibliographic Review

**Figure 1:**
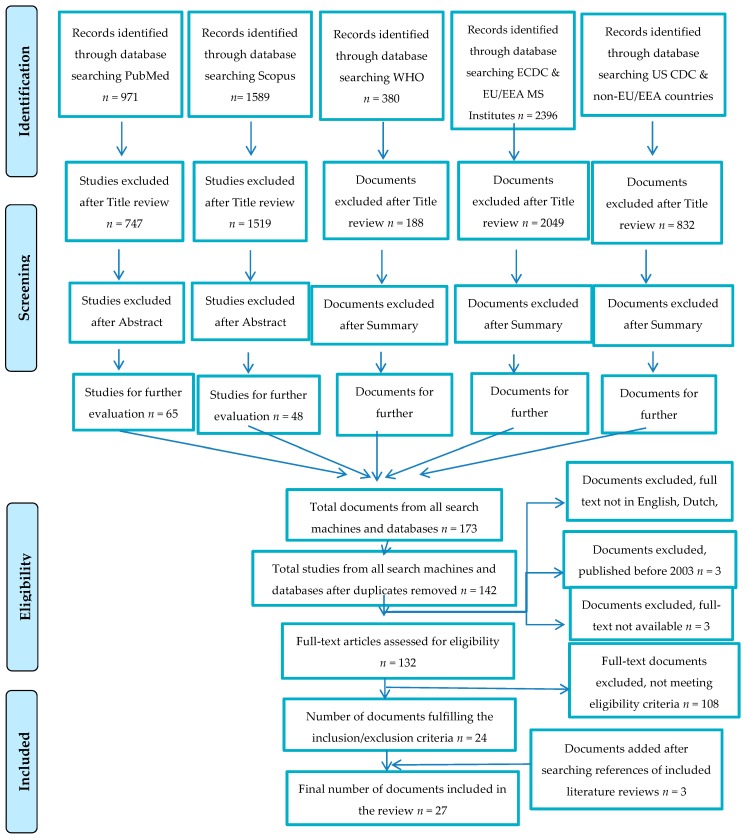
Flow diagram of study selection process.

After full-text review the eligibility criteria were fulfilled by 27 articles (24 identified through databases searched and three after checking the reference lists of full-text articles). Figure 1 presents the flow chart of the review process with articles retrieved, the number of articles excluded and the reason for exclusion, and finally the number of articles that fulfilled the inclusion criteria. 

The two researchers independently reviewed and extracted data from the 27 documents by using a standardized questionnaire (Appendix C). 

**Figure 2:**
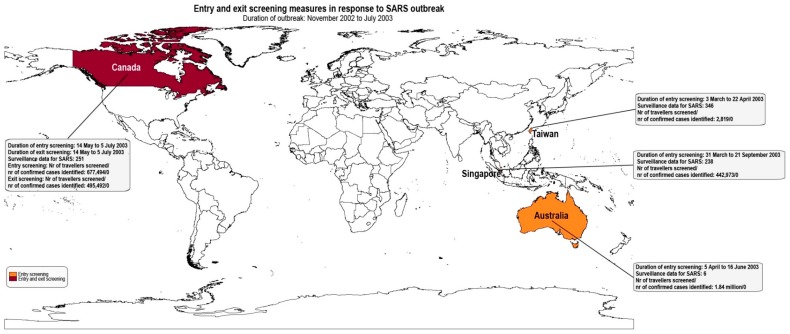
Published entry and exit screening measures in response to SARS outbreak [8,9,23,35,42].

**Figure 3:**
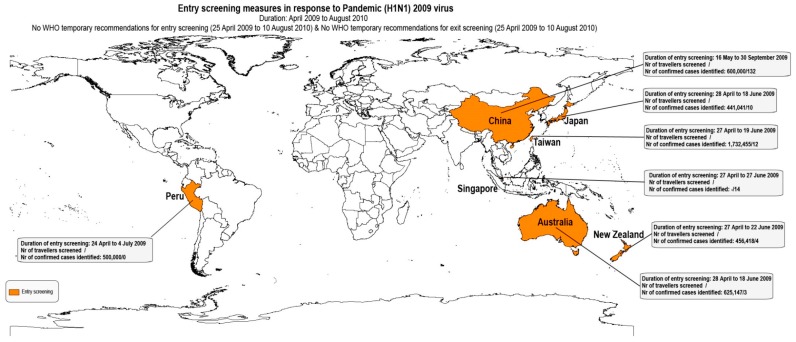
Published entry screening measures in response to Influenza Pandemic (H1N1) 2009 [20,21,24,32,33,36,40].

**Figure 4:**
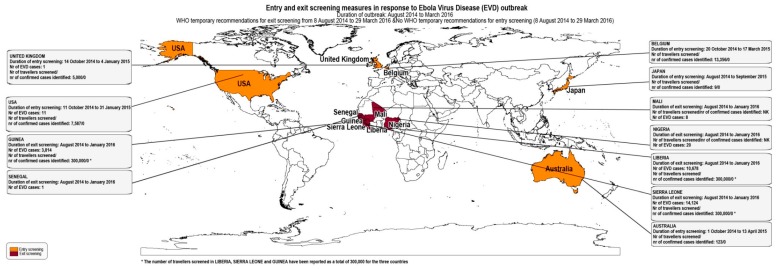
Published entry and exit screening measures in response to Ebola virus disease outbreak [16,27,31,34,38,41].

As shown in Table 4, from the 27 articles, 25 reported entry screening measures [6,7,8,9,10,11,15,20,21,23,24,26,27,28,29,31,32,33,34,35,36,37,38,39,40] and five reported exit screening measures [6,9,16,22,28]. Figures 2–4 summarize the results of entry and exit screening measures by disease and per country.

#### 3.2.1. Entry and Exit Screening Measures in the Different Types of Point of Entry

(1) Airports 

Australia implemented entry screening measures at airports to prevent EVD [31], Influenza Pandemic (H1N1) 2009 cases [20] and SARS [8]. New Zealand implemented entry screening for Influenza Pandemic (H1N1) 2009 at airports [21]. In Canada, entry and exit screening was applied for SARS [9] and entry screening for EVD at airports [22]. Peru implemented entry screening for Influenza Pandemic (H1N1) 2009 at airports [32]. In China, entry screening took place for Influenza Pandemic (H1N1) 2009 at airports [40]. In Guinea, Liberia, Sierra Leone, Nigeria, Senegal, and Mali, exit screening was implemented for EVD at airports, seaports and ground crossings [6,16,22,28]. Japan implemented entry screening for EVD [38] and Influenza Pandemic (H1N1) 2009 at airports [24,26,39]. Singapore applied entry screening for Influenza Pandemic (H1N1) 2009 at airports [36] and for SARS at airports, seaports and road entry points [23,29]. Taiwan applied entry screening at airports for SARS [35], Influenza Pandemic (H1N1) 2009 [33] and Zika virus disease [7] as an ad hoc measure at airports in response to public health emergencies. Moreover, entry screening for Dengue [7,10,11,15] and Chikungunya [7] were implemented as routine measures at airports. Regarding European countries, Belgium and United Kingdom implemented entry screening at airports for EVD [22,27,37]. Table 4 summarizes the entry and exit screening measures. 

(2) Ports 

Entry screening measures were implemented at seaports in four countries [8,23,27,29]. Exit screening measures were implemented at seaports for EVD in three counties [16]. A two-level program was applied at Australia’s seaports for SARS, where the Australian Quarantine and Inspection Service staff directly contacted the Chief Quarantine Officer to inform of ill passengers [8]. Temperature checks for SARS were introduced to all of Singapore’s ferry/sea terminals [23,29]. The public health authorities of Belgium implemented entry screening for EVD at seaports located in priority areas [27]. In Guinea, Liberia, and Sierra Leone exit screening for EVD at seaports included temperature checkpoints, followed by emergency medical response, on-site isolation and use of personal protective equipment for staff if necessary [16].

(3) Ground crossings 

Two articles [23,29] refer to entry screening measures for SARS implemented at road entry points, one [16] to exit screening measures for EVD at ground crossings, and two articles [27,37] refer to entry screening measures at a train station for EVD. Thermal scanners were installed at the road entry points of Singapore to check the temperatures of all departing and arriving passengers for SARS [23,29]. As reported by Cohen et al., in the land borders of Guinea, Liberia, and Sierra Leone, simple exit screening measures involving visual screening for illness at designated official border crossings were applied for EVD [16]. Due to sparse, understaffed, and under-resourced official border points of entry, land borders were characterized as “porous” and it was not possible to apply measures similar to airports. Two articles refer to entry screening measures implemented at the Eurostar terminal/train station at London St Pancras for EVD [22,37]. Measures included visual and fever screening.

#### 3.2.2. Timeframes of Public Health Events and Screening Measure implementation

Table 5 presents the timeframes of public health events, of screening measure implementation, and of temporary recommendations for screening measures issued by WHO. As presented in Table 5 Since the IHR 2005 entered into force in 2007, temporary recommendations for exit screening measures have been issued by WHO as part of a set of measures to be implemented in areas affected from outbreaks. This happened during the EVD epidemic in West Africa in 2014/2015, in the EVD outbreak in the (Democratic Republic Congo, DRC) in 2018 and during the plague outbreak in Madagascar in 2017 [5]. On the contrary, entry screening measures were not part of WHO temporary recommendations for the outbreaks of EVD in West Africa, Poliovirus, EVD outbreak in DRC, Middle East respiratory syndrome (MERS), Yellow fever, Zika virus disease, Plague and the Influenza Pandemic (H1N1) 2009 [5]. 

#### 3.2.3. Screening Measures on an Ad hoc Basis and as a Routine Measure

In Taiwan entry screening was applied as a response to public health emergencies as well as on a routine basis. In particular, entry screening was applied for Zika virus disease for a total of 10 months (January to October 2016) [7]. Moreover, entry screening measures in Taiwan are applied on a routine basis for vector-borne diseases. The articles fulfilling the inclusion criteria reported results for entry screening routine measures for Chikungunya infection between 2013 and 2016 [7]. Routine entry screening for all inbound travelers for Dengue has been implemented since 2003 and is ongoing [7,10,11,15].

Only Taiwan implemented entry screening on a routine basis for Dengue and Chikungunya [7,10,11,15]. All other authors reported screening measures on an ad hoc basis in response to an emergency public health event.

Targeted screening of incoming travelers arriving from affected countries was implemented in five countries for Influenza Pandemic (H1N1) 2009 [21,24,26,33,39]. Six countries implemented massive general screening to all inbound travelers arriving at the airport for SARS and for Influenza Pandemic (H1N1) 2009, as well as for Dengue fever and Chikungunya infection [8,9,10,15,20,29,32,35,36,40]. In one report the type of screening is not clearly described [28].

#### 3.2.4. Decision-Making

Screening measures for SARS in Canada were decided by Health Canada [9] and in Singapore by the Ministerial Committee on SARS chaired by the Minister for Home Affairs [29]. In Japan, concerning Influenza Pandemic (H1N1) 2009 and EVD, decisions were taken at the level of the Ministry of Health, Labor, and Welfare, while the response and measures of relevant ministries and agencies were coordinated at the Intergovernmental Coordination Meeting on EVD measures, chaired by the Deputy Chief Cabinet Secretary for Crisis Management [26,38,39]. In New Zealand, the Ministry of Health and the Auckland Regional Public Health Service [21] and in Taiwan, the Central Epidemic Command Center were responsible for decision-making regarding the Influenza Pandemic (H1N1) 2009, Zika virus disease and Dengue fever screening measures [7,15,33]. 

#### 3.2.5. Authorities Involved in Implementing Entry/Exit Screening Measures

Authorities and officers involved in the implementation of screening measures were ministries of health, public health officers/inspectors or public health emergency staff, custom, and border control staff, airlines, airport, and port authorities, emergency medical service units at airports/ports, airline check-in agents, flight crews, airport ambulance services, physicians, nurses, quarantine officers, regional authorities and communities and fire brigade [6,7,9,20,21,26].

#### 3.2.6. Contact Tracing, Data Management, and Communication Flows

Health Canada introduced a traveler contact information form that collected contact details and information on location of stay that all inbound passengers were asked to fill in before arrival, when implementing entry screening for SARS [9]. Upon landing, all forms were collected from passengers by Health Canada personnel and retained for possible contact tracing if a case was subsequently identified. The traveler contact information form is believed to have reduced the time for securing the manifest from weeks to two days.

During entry screening measures for EVD implemented in the US, contact information for inbound travelers from affected countries was entered into a database and transmitted to states through CDC’s Epidemic Information Exchange (Epi-X) that is a secure notification system [6]. As part of entry screening in Australia for EVD, the Notifiable Conditions Information Management System database was used [31]. 

In Taiwan, all data and diagnostic results of cases of Dengue fever identified through screening were reported through the web-based National Surveillance System, for later tracking and management of cases [10]. 

During entry and exit screening measures applied in Canada for SARS, Health Canada monitored the spread of SARS via the WHO-Health Canada Global Public Health Intelligence Network and regular communications with other international and Canadian provincial and territorial public health agencies documenting travel and illness histories of possible SARS cases who departed Canada and whose illnesses were diagnosed and reported internationally [9]. 

As part of entry screening measures in Taiwan for Influenza Pandemic (H1N1) 2009, information of suspected cases was delivered to the local health bureaus via the Internet Information System for subsequent follow-up [33]. 

In China information was entered into an internet-based surveillance system; all community hospitals were authorized to access the system [40]. 

#### 3.2.7. Screening Protocols and Accompanying Measures

Regarding the screening protocols applied, both entry and exit screening included an initial assessment of exposure through completion of a questionnaire, temperature measurement, and if needed, secondary assessment by medical staff and laboratory testing. Screening measures were conducted at the point of entry facilities. One article reported that primary screening measures were conducted on board the aircraft [26]. Some countries combined screening measures for symptoms and exposure with risk communication and instructions strategies, and by offering equipment for body temperature measurement (Table 6). 

As far as exit screening for EVD is concerned, visual screening, health questionnaires and temperature measurement (non-contact infrared thermometer) were applied [6,16,22,28]. 

Similarly, entry screening for EVD included screening of travelers by asking questions about symptoms and potential exposure risks and temperature checks [6,16,22,28]. Entry screening measures for EVD in the US were combined with an educational and informing strategy to travelers passing through the screening points [6]. Each traveler arriving from an affected country received a Check and Report Ebola (CARE) kit that included health education materials, a thermometer, and ways to connect with their state or local health department, including a prepaid cell phone [28]. In Australia, a separate EVD-specific arrivals card was distributed [31]. 

For exposure and/or symptoms assessment, travelers were asked to complete a questionnaire [6,7,9,10,11,16,20,22,23,26,27,29,31,35,37,38,39,40]. In addition to this, temperature measurements were conducted either with handheld non-contact infrared thermometers, or thermal imaging scanners [6,7,8,9,10,11,15,16,20,22,23,24,26,27,29,33,34,35,36,37,38,39,40]. One article referring to entry screening for Zika virus disease [7] describes that travelers underwent visual assessment, and infrared cameras were used to detect abnormal temperatures. An ear thermometer was used to recheck temperature when necessary.

Visual screening for the presence of symptoms was also part of the screening protocols [6,7,8,9,10,11,16,20,21,22,23,26,27,33,34,35,37,38,40] and/or rapid diagnostic tests [7,10,24,26,39] to identify suspected cases. Finally, medical and laboratory assessments were conducted for suspected travelers [6,7,8,9,10,11,15,16,20,21,22,23,24,26,27,29,31,32,33,34,35,36,38,39,40]. Methods for entry and exit screening, accompanying measures, response measures and laboratory diagnosis are summarized in Table 6.

In all but one article included in the literature review, entry screening to in-transit travelers has not been addressed. Those in-transit travelers were screened during entry screening measures for Influenza Pandemic (H1N1) 2009 at the airport in Japan, either at the aircraft cabin or at the quarantine station at the terminal [39].

#### 3.2.8. Technology for Body Temperature Measurement

Temperature measuring devices used to measure body temperature of travelers were electronic handheld or fixed/stationary non-contact thermometers, and ear or oral thermometers [6,7,8,9,10,11,15,16,20,22,24,26,28,29,33,35,36,39]. The specific model for temperature measuring devices was described in three articles: Flir A40 or Flir P20 [10] and TVS-500EX, (NEC Avio Infrared Technologies Co., Ltd., Tokyo, Japan) [24,39]. Non-contact thermometers were used mainly in primary screening, and contact or minimal contact thermometers in secondary screening.

Screening protocols for SARS in Canada, Taiwan, and Singapore used thermal scanning machines as part of the primary screening, while as part of the secondary screening, Australia and Taiwan used ear temperature thermometers and Canada used oral temperature thermometers [8,9,35]. 

Screening measures implemented in response to Influenza Pandemic (H1N1) 2009 in Australia, Japan, Singapore and Taiwan were conducted using stationary infrared thermoscanners [20,24,26,33,36,39]. In Japan ear or axillary temperature measuring devices were also used [24]. Entry primary screening temperature measurements in the US and exit primary screening temperature measurements in Guinea, Liberia, and Sierra Leone were conducted with handheld NCITs [6,22].

#### 3.2.9. Appraisal of Impact of Entry and Exit Screening Measures Based on Case Identification

Entry screening measures for SARS did not identify any confirmed cases in the studies included in this review; however, cases of SARS were notified in the countries where screening took place. Entry and exit screening measures for EVD did not identify any confirmed cases. In the two (United States, United Kingdom) out of the five countries that implemented entry screening (Australia, Japan, United States, Belgium, United Kingdom), EVD disease cases were imported (one case in the UK and nine in the US), but were asymptomatic during travel [27,34]. The detection rate of confirmed Influenza Pandemic (H1N1) 2009 cases among all passengers screened ranged from 2.2 to 0.01 per 10,000 travelers in China and Japan, respectively [24,40].

The numbers of travelers screened and identified as suspected and confirmed can be found in Table 7 for entry screening and in Table 8 for exit screening. Table 7 and Table 8 include surveillance data of cases from countries that implemented entry/exit screening for the infectious diseases [15,41,42,43,44,45,46,47].

For SARS, six out of the 46 suspected cases identified through entry screening measures for SARS were diagnosed with atypical pneumonia or chronic obstructive pulmonary disease with secondary infection or bronchopneumonia in Taiwan [35]. SARS was not confirmed in any suspected cases. 

In Australia, four hospitalized persons were ultimately given an alternative or undetermined diagnosis other than the initial targeted SARS [8]. 

For Influenza Pandemic (H1N1) 2009, Among 391 travelers identified as suspect cases during entry screening measures applied for Influenza Pandemic (H1N1) 2009 in Japan, five were diagnosed as influenza type A and one as type B [26]; while genotyping showed that among the five type A cases, one was Russian flu (H1N1), one was Hong Kong flu (H3N2), and three were Influenza Pandemic (H1N1) 2009. 

Similarly, two other articles [24,39] described cases that were finally diagnosed with Influenza type A or B, and not the Influenza Pandemic (H1N1) 2009 that screening targeted in Japan. 

For EVD, during entry screening measures applied for EVD in Australia, six out of 123 screened travelers from EVD-affected countries developed symptoms compatible with EVD, and when further assessed, were diagnosed with influenza or upper respiratory tract infection [31]. Entry screening measures applied for EVD in Japan identified nine individuals with compatible symptoms, who were finally diagnosed with malaria (four travelers), influenza (four travelers) and other (one traveler) [38]. In an article describing entry screening procedures for Zika virus disease in Taiwan, five out of 21,083,404 inbound screened passengers were laboratory-confirmed cases of Zika virus disease; whereas 130 cases of Dengue fever and four cases of Chikungunya infection were found [7].

None of the 27 articles fulfilling the eligibility criteria systematically conducted a cost-effectiveness analysis for entry or exit screening measures. Five articles addressed issues for the cost of screening measures (Table 9) [9,10,15,20,23].

#### 3.2.10. Management of Suspected Cases

Table 6 summarizes the case management where available. Concerning the applied protocol after diagnosis and management of cases of Influenza Pandemic (H1N1) 2009, two articles described how patients positive for H1N1 were hospitalized/isolated, and close contacts or persons seated within 2 m around the index patient during the flight were quarantined [26,39]. One article described that persons tested positive to H1N1 with RT-PCR were offered oseltamivir, and were sent home or to a facility for isolation [21]. The ECDC report describes the management of treating EVD patients in designated hospitals, including isolation, personal protective equipment, samples, waste management and post-mortem procedures [27].

In respect to health measures applied to the travelers during the exit screening for EVD, the ECDC technical report refers that 77 out of 36,000 screened travelers were identified and denied boarding, although none were later diagnosed with EVD [22]. 

Another ECDC report concerning entry screening for EVD, describes that in the UK persons in the low and higher risk exposure categories were monitored for 21 days after leaving the country of interest by public health emergency services [27]. 

The Health Canada’s protocols for airplane passenger contact tracing had evolved during the SARS outbreak and were updated during the Influenza Pandemic (H1N1) 2009 [9]. At the beginning, contact tracing of passengers included follow-up of passengers seated in the same row, two rows in front, and two rows behind someone with a probable case, who was symptomatic while in flight. Later, contact tracing was expanded to include persons who were contacts to suspected cases while in flight. In Japan, local authorities received contact information about overseas travelers from the competent authority at the airport and monitored their health daily by phone. Later on, the observation by local authorities was performed for seven days only for those seated within two meters from a patient [26]. 

During the implementation of entry screening measures for Influenza Pandemic (H1N1) 2009 in Japan, patients positive for H1N1 were isolated, and close contacts were quarantined [39]. At first, health monitoring by health centers was performed for passengers arriving from affected countries, and later for only those who had come into contact with the individuals identified by entry screening. Enhanced surveillance included mandatory reporting of details of the infected individuals. An entry card was given to all arriving passengers instructing them to consult with staff at public health centers in the event of developing symptoms while in Japan. 

In another article [40] general practitioners at the community hospitals performed medical follow-up on the foreign travelers, who were contacted daily for 7 days after entry into Beijing by the general practitioners by telephone or face-to-face interview, in order to report on their health status. When a traveler reported having influenza-like illness symptoms, she/he was asked to attend the jurisdictional hospital for testing.

#### 3.2.11. Limitations of Screening Measures and Challenges Reported

Regarding challenges, 11 out of 26 articles refer to limitations, failures, and mishaps of applied screening measures. 

Samaan et al. noted that the applied screening measures may still have been ineffective due to false declarations by travelers, denying contact with people with SARS, or taking antipyretic drugs to conceal fever [8]. Lee et al. mentioned that travelers tried to conceal symptoms so as to be treated in Taiwan where medical fees were lower than in Hong Kong during the SARS outbreak (Figure 2) [35]. St John et al. highlighted that screening measures (health alert questionnaires and thermal scanning machines) were non-specific for SARS [9]. 

Regarding screening measures for Influenza Pandemic (H1N1) 2009 (Figure 3), Gunaratnam et al. referred to the underestimation in the number of cases acquired overseas [20]. Fujita et al. noted that during the incubation period, when patients have no symptoms or high fever, it is almost impossible to identify patients by quarantine officers, coupled with the quick inspection kit having only about 70% accuracy [26]. Similarly, Hale et al. attributed ineffectiveness (estimated sensitivity 5.8%) of screening measures to the high proportion of asymptomatic infected travelers, incubation of infections acquired before or during a flight, reliance on self-identification, limitations of case definitions, and limitations of thermal scanning [21]. 

The authors of the 27 articles fulfilling the eligibility criteria of the current review attempted to assess the impact of screening measures or commented on the impact, as presented in Table 10. Assessment of public health impact.

In Appendix F, WHO statements (concerning screening and travel restrictions in relation to public health events) that were used in the current report are presented.

The screening methods protocol design and the robustness of application can influence the effectiveness of screening measures [50]. The exposure and symptom assessment methods, the tools and type of equipment used, and the number of staff involved and their training play an important role in the outcome of screening measures [50]. Screening protocols for symptoms and exposure assessment included questionnaires, health alert leaflets, visual checks, and body temperature measurements. Each method has its strengths and limitations [22]. In the reviewed evidence, self-reporting of exposure and symptoms in questionnaires relies on the honesty of the responder, language barriers exist, and fever symptoms can be concealed by antipyretic drug use [8]. Screening cannot detect incubating or asymptomatic travelers [24]. Fever and other symptoms are non-specific and planning and resources are needed for possible high demand of laboratory tests [22]. During the EVD epidemic in West Africa in 2014/2015, (Figure 4) some travelers attempted to escape entry screening by presenting passports which did not show that they had traveled to an affected country [51]. In general, the prevalence of disease targeted by screening is very low among travelers, and the positive predictive values and the sensitivity of screening measures are expected to be very low for the diseases targeted through screening [9]. This is expected especially when general massive (to all travelers) rather than targeted screening measures (e.g., to travelers coming from affected countries or certain direct flights) are applied.

Data from the systematic bibliographic review were used to develop the algorithm for making evidence-based decisions in implementing entry and exit screening measures (Appendix E). 

## 4. Discussion

### 4.1. Impact of Exit Screening Measures at Airports, Ports, and Ground Crossings

Evidence from this review about prevention of international transmission of disease by detecting and prohibiting travel to exposed or ill travelers from affected countries is mainly based on the measures implemented in response to EVD in Guinea, Liberia, Sierra Leone, Nigeria, Senegal, and Mali [6,16,28,49]. In total, about 300,000 were screened in Guinea, Liberia, and Sierra Leone, but no case was detected through exit screening measures. During the study reporting period, four confirmed cases were exported through air travel during the exit screening measure implementation, but were not symptomatic while traveling [16]. EVD is a disease with high pathogenicity and a very low number of asymptomatic cases [52]. Consequently, it is probably unlikely that additional cases would have been exported through air or sea travel without being detected by surveillance systems in the destination non-affected countries. 

An assessment of the impact of exit screening measures at ground crossings is much more challenging than at airports and seaports. The 2014/2015 Ebola outbreak spread through the population movement in the land borders of Guinea, Liberia, and Sierra Leone and was later introduced to Senegal and Mali [16]. As reported by Cohen et al., the implementation of land-border screening measures was challenging given the high mobility of populations through formal and informal points [16]. It was not possible to apply at ground crossings the same protocols that were applied at airports and seaports. Exit screening at ground crossings combined visual checks for symptomatic persons at official ground crossings, health education, and community engagement, as well as implementation of plans for isolation, communication, assessment, referral, and transportation. To the best of our knowledge, there is no published evidence meeting the inclusion/search criteria about the positive impact of screening measures at ground crossings in preventing the exportation of EVD cases from affected countries. 

Exit screening measures may have helped to prevent extensive travel and trade restrictions, by providing confidence to the different stakeholders that measures are in place to protect the public from exportation of cases. As stated by Rhymer and Speare, travel and trade restrictions disregarding WHO recommendations were implemented worldwide in 58 (31.0%) of 187 WHO State Parties [53]. Exit screening measures may have balanced the overreactions. Exit screening measures enabled business continuity to trade and transport sectors, as well as the continuation of public health organizations and humanitarian missions to support the affected countries [16,27,28]. Another secondary effect of screening measures is that thorough exit screening measures at borders may have played a role in discouraging ill or exposed persons from attempting to leave the affected countries [25]. 

It is unknown how many cases would have been exported if exit screening measures at the points of entry of the affected countries would not have been implemented. Even if no case was detected through exit screening measures, concomitant benefits from exit screening measures may be of paramount importance and should also be considered when assessing impact and making decisions for health measures. Considering that all countries should be prepared to deal with unexpected events as laid down in the IHR 2005, and lessons learnt are available from past events for which temporary recommendations for exit screening measures were issued by WHO, all countries should have the capacities to implement exit screening measures at points of entry (designated airports, ports, and ground crossings), and this should be part of the preparedness planning [3,5].

### 4.2. Impact of Entry Screening Measures

Since the IHR 2005 entered into force in 2007, temporary recommendations for exit screening measures have been issued by WHO as part of a set of measures to be implemented in areas affected from outbreaks [5]. On the contrary, entry screening was recommended only for specific settings and timeframes by WHO, and only in response to the Ebola outbreak in DRC in 2018 [5]. Moreover, advice was given that if entry screening measures are implemented, countries should consider that “*entry screening may have a limited effect in reducing international spread when added to exit screening, and its advantages and disadvantages should be carefully considered… if entry screening is implemented, States should take into account the following considerations: it offers an opportunity for individual sensitization, but the resource demands may be significant, even if screening is targeted; and management systems must be in place to care for travelers and suspected cases in compliance with International Health Regulations (IHR) requirements*” [5]. 

The primary objective of entry screening measures is to prevent or to delay introduction of ill or incubating cases to a country. Evidence for achieving this primary objective is based on the measures implemented in response to the EVD epidemic in West Africa in 2014/2015, SARS, Influenza Pandemic (H1N1) 2009 and Zika virus disease, as well as entry screening measures implemented on a routine basis for Dengue fever and Chikungunya infection. None of the countries that implemented entry screening for SARS detected any case [8,9,23,29,35]. For Influenza Pandemic (H1N1) 2009 the detection rate ranged from 0.01 to 2.2 confirmed cases per 10,000 persons screened [20,21,24,26,32,33,39,40]. A survey conducted by WHO showed an aggregate rate of 4 confirmed cases per 1,000,000 screened travelers for Influenza Pandemic (H1N1) 2009 in 10 countries [50]. For EVD, no case was identified through entry screening measures [6,27,28,31,34,37,38]. For Zika virus disease, five cases were identified and more than 21,000,000 persons screened [7]. Routine entry screening measures for Dengue fever showed a detection rate of less than 8%. It should be noted that the diseases targeted by entry screenings such as SARS, EVD and the Influenza Pandemic (H1N1) 2009 have a very low prevalence among travelers, therefore the positive predictive value of entry screening is expected to be close to zero [9]. Cowling and colleagues compared the dates of the first reported case of Influenza Pandemic (H1N1) 2009 in countries which implemented entry screening measures with countries that did not implement such measures. They concluded that entry screening may delay the introduction of a new influenza strain for about 7–12 days [54]. However, it should be noted that it was not possible for the study to assess which measures other than entry screening implemented by the countries have contributed to delaying introduction. 

Several attempts were made by researchers to evaluate the public health impact of entry screening measures by comparing numbers of cases identified through screenings at airports with the total number of imported cases, or with cases locally acquired in the country in the same timeframe. However, the onset of symptoms was not assessed in all cases and it is not clear if the imported cases passed through screening were symptomatic or incubating. Entry screening at airports implemented on routine basis proved to be successful in Taiwan (an island) in identifying about half of the imported cases of Dengue fever [7,10]. Twelve out of 59 imported cases of Influenza Pandemic (H1N1) 2009 were detected through entry screening within 54 days of entry screening [33]. In Japan, 6.6% (10/151) of Influenza Pandemic (H1N1) 2009 cases were identified by airport entry screening [39]. Another study in Japan showed that only 11 confirmed cases of Influenza Pandemic (H1N1) 2009 were detected through entry screening, but 633 cases were diagnosed among the Japanese population and about 20% of them had passed through the entry screening [26]. The detection rate of Dengue fever and Chikungunya infection was higher than that of influenza. This can be attributed to the difference in the severity of symptoms and whether it can be observable or measurable when passing the entry points at airports, as well as the rate of persons who will ask for medical care in the health care system and will be captured by the routine surveillance system. Moreover, this can be attribute to the fact that entry screening for vector-borne diseases in Taiwan has been implemented on a routine basis for long periods of time and not as part of response measures to emergencies that are implemented in short periods of time. 

According to the results of this review, evidence suggested that the primary objective of entry screening implemented in response to public health emergencies—which is to detect imported cases at borders—was not achieved, but several beneficial concomitant effects have been reported in several instances, including educating travelers passing through the screening points, providing contacts of public health authorities to travelers in case they develop symptoms, collecting contact details for contact tracing, maintaining confidence that air travel is safe, preserving public confidence, and helping to avoid major economic, social and international impacts which even a single imported severe disease can cause [6,23,25]. Entry screening alone seems to be ineffective in preventing or delaying introduction of diseases to a country; however, it could be justified for severe diseases, as part of a set of measures complementing each other, after setting priorities and where there are available resources [26]. The ECDC suggests that entry screening at airports in combination with exit screening could be of value, if exit screening measures are questionable and if the timeframe between departure and arrival at the destination country is long [22]. 

The research strategy of this bibliographic review did not reveal statistical data specifically for entry screening at ground crossings. Crossing land borders for sick, symptomatic persons may be easier than moving through air or sea means of transport. The density of populations crossing land borders can be very intense or not at all from place to place. The EVD epidemic in West Africa in 2014/2015 spread between the affected countries in West Africa through land borders [16]. This fact should be considered by policy makers for preparedness planning. Countries where many official and unofficial crossing points exist or countries where border checks are not routinely conducted at ground crossings may not be well prepared to respond. Preparedness activities for potential unexpected events should include plans for implementation of screening measures at ground crossings. Screening measures at land borders require cooperation among neighboring countries and regions. However, considering the previously reported challenges and the lack of evidence on the impact of entry screening measures at ground crossings, this area may represent a gap in preparedness in the event of a high risk of exportation/introduction and spread of disease through land borders of neighboring countries. 

### 4.3. Cost-Effectiveness of Screening Measures

Very limited information is available about the cost and cost-effectiveness of screening measures. General entry screening measures at airports of Australia for Influenza Pandemic (H1N1) 2009, Canada for SARS, and Taiwan for Dengue fever cost about US$50,000 per case detected (airport clinic staffing cost), a total of 7.55 million Can$ investment for a period of four months, and US$ 43,000 for each set of instruments used at screening, respectively [10]. Investing in screening measures reduces the resources from other possibly more effective measures [9,23]. Further cost-effectiveness studies could be conducted to analyze the cost and benefits of screening measures, and to compare these with other public health measures to inform decision-makers. 

### 4.4. Decision-Making

Although the inability of entry screening measures to identify cases of SARS in the 2003 outbreak was known during the public health emergencies that occurred the following years, decision-making during the Influenza Pandemic (H1N1) 2009 and the EVD epidemic in West Africa in 2014/2015 seemed to be based on other reasons. Several authors suggest that screening measures in several cases may have been implemented mainly to relieve political and social pressure, and limit negative economic consequences from travel and trade restrictions [8], as well as to preserve public confidence [8,9,29] and maintain confidence that air travel is safe [6]. 

When temporary recommendations from WHO for screening measures have been issued in response to a public health event, then countries should have the capacities to implement such measures. Other factors of consideration are: the disease severity, the transmissibility (and whether the transmission takes place before or after symptom onset), the mode of transmission, the incubation period, the symptomatology of disease, and how easily the disease can be detected, the proportion of febrile patients, the temporal and spatial extent and the phase of the outbreak, any available results from modeling studies, the type of country, and point of entry, the disease epidemiology in the country, the volume of travelers and connections to affected areas (Appendix E). All these factors should be considered to estimate the possible expected detection rates, and to balance this with other measures that could be implemented, and the cost and effectiveness of those. A mathematical model presented by Gostic, K.M., and colleagues, demonstrated how different factors of six disease and outbreak characteristics and human behavior can affect screening measures’ effectiveness. It showed that “for pathogens with longer incubation periods, exposure risk detection dominates in growing epidemics, while fever becomes a better target in stable or declining epidemics. For pathogens with short incubation, fever screening drives detection in any epidemic stage. However, even in the most optimistic scenario arrival screening will miss the majority of cases” [18].

### 4.5. Modeling

The scoping search identified several studies reporting results of modeling that can inform decision-makers about the potential effects of entry screening measure implementation. A study modeled the number of expected Ebola infected travelers exiting affected countries, the potential effect of air travel restrictions, and the efficiency of airport-based traveler screening at international points of entry and exit [55]. The study concluded that exit screening measures at three airports in the affected countries would be successful in assessing all potentially exposed or Ebola-affected travelers. Another study created a model to assess the effectiveness of entry screening for the 2009 Influenza Pandemic (H1N1) in the US and concluded that it will not significantly delay arrival of influenza cases by air travel, but will reduce the rate of new US cases and subsequent deaths [56].

### 4.6. Resources and Logistics

If entry or exit screening measures are decided to be implemented, detailed planning is required, with careful execution to ensure consistent application by all staff involved and to all targeted travelers. The timing (starting and stopping of screening measures), the screening methods, the technology and tools, the human resources and training issues should be considered in the preparedness and response plans. Training of staff is an important component and should address recognizing the signs and symptoms of the disease, screening procedures and documentation, and appropriate use of personal protective equipment and technology for measuring body temperature [6]. 

Interview space must be available at the facilities of the point of entry as required by the IHR 2005 [3]. The WHO suggests that preparedness plans’ functionality should be periodically tested with simulation exercises [57]. Standard Operating Procedures (SOPs) for entry or exit screening measures could be tested in practice with simulation exercises. Documented, regularly updated and tested national guidelines and SOPs for health measures at points of entry, including entry and exit screening, are checked in the framework of the joint external evaluation of core capacities [58]. Capacities for entry and exit screening should be part of the national planning. 

The most suitable site of primary and secondary screening should be decided: on board the conveyance, at the terminal, or before or after checking in or collecting luggage. Gaber and colleagues suggest that exit screening at airports should take place before travelers deliver luggage at the terminal, to avoid the checking-in of suitcases from infected travelers that later may need to be traced and removed [59]. 

Further essential resources include capacities for laboratory diagnosis, quarantine, isolation, and treatment of suspected exposed or affected travelers. In the US, during the EVD epidemic in West Africa in 2014/2015 customs and border protection officers conducted the primary entry screening at airports and public health officers conducted the secondary screening [6]. As suggested previously, entry screening should be part of a broader set of measures and different stakeholders need to cooperate. Both the public and the private sectors, the transport industry, points of entry administrations and actors at all levels, from the local point of entry to the national, EU and international level should be involved [7]. Guidance and advice entry and exit screening measures from international organizations may further support decision-making. 

Other issues for consideration about entry screening are the identification of targeted travelers or itineraries at ports, airports, and ground crossings, including lists of returning workers from missions in affected countries (if applicable, obtained from aid recruiting organizations), lists of visas granted to affected countries, disclosure policies, and expert support on legal, communication, health advisory and others issues [31,38]. 

This bibliographic review showed that most of the entry primary fever screening for SARS, Influenza Pandemic (H1N1) 2009 and vector-borne diseases was conducted using thermal scanner cameras, followed when necessary by secondary screening using NCITs or contact thermometers. An ECDC technical report reviewed evidence about the accuracy of body temperature measuring devices and concluded that there are a variety of technologies available commercially; some NCITs are approved for use as diagnostic tools as happens with the contact thermometers, but thermal scanner cameras have not been evaluated for such purpose [22]. The report continues that NCITs are more accurate than the thermal scanner cameras. The US CDC evaluated the performance of NCITs, showing a sensitivity of 80–99% and a specificity of 75–99%. Equipment calibration and accuracy checks according to manufacturers’ instructions, and training of staff in the correct use are essential during screening measure implementation. 

### 4.7. Limitations of the Bibliographic Review

Research questions of the bibliographic review were answered based on published information; much more unpublished evidence may exist that could not be considered. Many other countries had implemented entry screening measures in response to the 2009 Influenza Pandemic (H1N1) on the European region, but very few of them published screening results and experience [50]. Databases searched index health-related publications; it is possible that additional publications exist related to cost-effectiveness of screening measures. Language bias could be one limitation of the review, since only articles in English, Dutch, German, and Greek were included in the review. In total, four articles were excluded due to language. Moreover, bias could occur from the fact that most of the published literature is about entry screening measures, rather than exit screening.

Screening measures to migrants, refugees, and asylum seekers were not under the scope of this review. In case of forced migration travelers often cross borders through unofficial points (land crossings or arriving by boats at coastlines) and not the official points of entry where authorities and structures exists. In this case, the authorities that are involved in screening and the procedures are different from what is applied at the official points of entry for regular travelers. Moreover, if migrants arrive in a country at the official points of entry, each country implements its own policy in terms of targeted diseases and examinations. This review did not analyze data from screening measures to migrants. 

## 5. Conclusions

For preparedness purposes and to be ready to respond to any unexpected public health event, all countries should have the capacities to implement entry and exit screening at designated ports, airports, and ground crossings. Exit screening measures could be prioritized compared to entry measures, based on past temporary recommendations issued during PHEIC. Evidence from this review suggests that entry screening measures alone are not effective in detecting imported cases at borders, but may allow opportunities for raising awareness and educating the traveling public. The current review further suggests that there are difficulties in assessing the impact of border screening measures. Statistical data demonstrate very low detection rates of cases in both entry and exit screening. The decision about the implementation of screening measures should be examined on a case-by-case basis, after considering the disease and outbreak characteristics, the country situation, and the available resources, which can be compared to the cost and effectiveness of other alternative measures. Screening measures have important concomitant effects when implemented in combination with health education and informative strategies for travelers, the decision-making process should take those effects into consideration. Specificities at each type of point of entry (port, airport, ground crossing) should be considered for the implementation of screening measures, since different approaches are needed for each type of point of entry. The implementation of entry and exit screening measures require planning, allocation of resources, and careful design and application of protocols. Decision-makers should be aware and consider the limitations of screening methods, including false declarations by passengers about exposure and disease signs and symptoms, use of antipyretic drugs to conceal fever by travelers, inability to detect incubating or asymptomatic travelers, language barriers, and false positive and false negative results expected from temperature measuring devices. Based on review results, an algorithm about decision-making for entry/exit screening was developed. Guidance and advice on decision-making related to entry and exit screening measures from international organizations would be helpful to countries when developing their preparedness plans, as well as when deciding about response measures to public health events. Training of staff are among the key issues for implementing a robust screening program at points of entry. 

## Figures and Tables

**Table 1:** Search terms.

Public Health Event	Type of Measure	Population of Interest (Humans)	Setting
Biological Biochemical Bacteriological Viral Microbiological Pathogen Public health risk Public health hazard Public health danger Hygiene Threat Exposure Pandemic Epidemic SARS H1N1 Flu Ebola Zika Plague Disease Influenza Infection Infectious Contagious Contagion Contamination Sick Sickness Illness Ailment	Exit/entry screening Entry/exit screening Entry and exit screening Exit and entry screening Entry screening Exit screening Border measure Border control Health assessment Health check	Patient Ill Sick Unhealthy Unwell Infected Affected Exposed Symptomatic Case Human Person Individual People Consumer Client Passenger Traveler Traveler Crew Refugee Migrant Immigrant Emigrant	Departure Exodus Debarkation Decampment Gateway Passageway Arrival Embarkation Checkpoint Airport Aerodrome Airdrome Air station Air terminal Flight terminal Aviation terminal Airfield Landing field Landing place Seaport Port Harbor Harbour Dock Pier Marine terminal Anchorage Port of embarkation Rail terminal Bus terminal Taxi (van) Ground crossing Land crossing Land-crossing Border crossing Frontier Terminal
(Biological OR Biochemical OR Bacteriological OR Viral OR Microbiological OR Pathogen OR Public health risk OR Public health hazard OR Public health danger OR Hygiene OR Threat OR Exposure OR Pandemic OR Epidemic OR SARS OR H1N1 OR Flu OR Ebola OR Zika OR Plague OR Disease OR Influenza OR Infection OR Infectious OR Contagious OR Contagion OR Contamination OR Sick OR Sickness OR Illness OR Ailment) AND (Exit/entry screening OR Entry/exit screening OR Entry screening OR Exit screening OR Entry and exit screening OR Exit and Entry screening OR Border measure OR Border control OR Health assessment OR Health check) AND (Patient OR Ill OR Sick OR Unhealthy OR Unwell OR Infected OR Affected OR Exposed OR Symptomatic OR Case OR Human OR Person OR Individual OR People OR Consumer OR Client OR Passenger OR Traveler OR Traveler OR Crew OR Refugee OR Migrant OR Immigrant OR Emigrant) AND (Departure OR Exodus OR Debarkation OR Decampment OR Gateway OR Passageway OR Arrival OR Embarkation OR Checkpoint OR Airport OR Aerodrome OR Airdrome OR Air station OR Air terminal OR Flight terminal OR Aviation terminal OR Airfield OR Landing field OR Landing place OR Seaport OR Port OR Harbor OR Harbour OR Dock OR Pier OR Marine terminal OR Anchorage OR Port of embarkation OR Rail terminal OR Bus terminal OR Taxi OR Ground crossing OR Land crossing OR Land-crossing OR Border crossing OR Frontier OR Terminal)			

**Table 2:** Inclusion and exclusion criteria.

**Inclusion Criteria**
1. Articles or reports or other documents published in peer-reviewed journals or national or international organizations’ publications, from January 2003 until May 2018 that report practices, implementation of guidelines, experiences, structures, processes, evaluation results about national routine or ad hoc entry or exit screening activities referring to travelers at ports or airports or ground crossings, worldwide.
2. Moreover, articles and documents that include information referring to any of the following (a) to (j) were included in the literature review: Type of screening (entry, exit) Type of infectious disease or diseases that entry or exit screening was targeting Type of points of entry (airports, ports, ground crossings) where measures were implemented Screening carried out on a routine basis or on an ad hoc basis after a public health event has occurred and its purpose Description of methods used in entry/exit screening (primary/secondary, questionnaire, body temperature checks, technology used) Type of technology used (thermometers, scan cameras, or other technology) After screening, the applied diagnosis protocol (laboratory and clinical examination) Number of cases identified, and total numbers of travelers screened for a specific timeframe, percentage of persons positive to screening that were diagnosed with the targeted disease, and percentage of persons diagnosed with a different disease from the initially targeted for the specific timeframe (positive and negative predictive values). General massive screening or targeted screening (e.g., traveler from any affected outbound country, all travelers directly arriving only from affected countries/areas, nationality of travelers, travelers in-transit that have called an affected country/area) Reporting on the assessment of the public health impact of the entry/exit screening measures or the cost-effectiveness of methods applied
Exclusion criteria
Articles that refer to migrants, refugees, and asylum seekers were excluded, except when related to a global health emergency response. Articles that refer to screening of diseases such as HIV and tuberculosis that were not part of a global health emergency response. Articles that described entry or exit screening measures that were part of response to a specific outbreak on board an airplane or a ship and not part of a country response to a global health threat. Articles for which the full text is not available in English, German, Dutch, or Greek were also excluded, unless the abstract clearly provided the information needed for data extraction.

**Table 4:** Summary results of studies reviewed including type of screening, point of entry (airports, ports, ground crossings), infectious disease targeted, and country of measure implementation.

Disease		Country	Type of Screening		Type of Point of Entry		
			Entry	Exit	Airports	Seaports	Ground Crossings
Respiratory infections	Severe Acute Respiratory Syndrome	Australia [8]	X	-	X	X	-
		Singapore [23,29]	X	-	X	X	X
		Taiwan [35]	X	-	X	-	-
		Canada [9]	X	X	X	-	-
	Influenza Pandemic (H1N1) 2009	Australia [20], China [40], Japan [24,26,39], New Zealand [21], Peru [32], Singapore [36], Taiwan [33]	X	-	X	-	-
Vector-borne diseases	Dengue fever *	Taiwan [7,10,11,15]	X	-	X	-	-
	Zika virus disease	Taiwan [7]	X	-	X	-	-
	Chikungunya infection *	Taiwan [7]	X	-	X	-	-
Other	Ebola virus disease	Australia [31], Japan [38], United States [6], Canada [22]	X	-	X	-	-
		Belgium [22,27]	X	-	X	X	-
		United Kingdom [22,27,37]	X	-	X	X	X
		Guinea, Liberia, Sierra Leone [6,16,22,28]	-	X	X	X	X
		Nigeria, Senegal, Mali [6,16,22,28]	-	X	X	-	-

* Screening measures for Dengue fever and Chikungunya infection were implemented as part of routine, long-term public health measures. All other screening measures were implemented on an ad hoc basis in response to public health emergency events.

**Table 5:** WHO temporary recommendations for entry and exit screening at points of entry (data in the table were extracted from the reports available from the WHO IHR (see Appendix F)).

Public Health Event			WHO Emergency Committee	WHO Temporary Recommendations	
Title	Started/Ended	PHEIC Yes/No (Date)			
				Entry/Exit Screening (Timeframe of Implementation)	On Travel Restrictions (Timeframe of Implementation)
Ebola outbreak in Democratic Republic of the Congo (DRC)	10 May 2018/25 July 2018	No	IHR Emergency Committee regarding the Ebola outbreak in 2018	Exit screening at airports (Mbandaka, Kinshasa), ports on the Congo river and congregation sites (23/5 to 25/7/2018)	Νo international travel or trade restrictions (10/5/2018 to 25/7/2018)
	4 August 2018/ongoing	No		Exit screening at defined points of entry in DRC (14/8/2018, ongoing, latest report on 5/12/2018)	Νo international travel or trade restrictions (4/8/2018, ongoing, latest report on 5/12/2018)
Ebola virus disease outbreak in West Africa	8 August 2014/29 March 2016	Yes (8/8/2014)	2014–2016 IHR Emergency Committee for Ebola virus disease	Exit screening in affected countries *, at international airports, seaports and major land crossings (8/8/2014 to 29/3/2016)	No general ban on international travel (8/8/2014 to 18/12/2015)
					No restrictions on travel and trade with Guinea, Liberia, and Sierra Leone (29/3/2016)
MERS	9 July 2013/ongoing, latest report on 3 September 2015	No	IHR Emergency Committee concerning Middle East respiratory syndrome coronavirus	No	Νo international travel or trade restrictions (17/6/2015, ongoing, latest report on 3/9/2015)
Influenza Pandemic (H1N1) 2009	25 April 2009/10 August 2010	Yes (25/4/2009)	IHR Emergency Committee concerning Influenza Pandemic (H1N1) 2009	No	Countries should not close borders or restrict international traffic and trade; If ill, it is prudent to delay international travel (if ill after travel seek care) (25/4/2009 to 10/8/2010)
Plague	4 October 2017/4 December 2017	No	WHO Regional Office for Africa	No	Νo international travel or trade restrictions (4/10/2017)
				Exit screening at International Airport in Antananarivo, Madagascar (9/10 to 4/12/2017)	Νo international travel or trade restrictions (4/10/2017 to 4/12/2017)
Poliomyelitis	5 May 2014/ongoing	Yes (5/5/2014)	IHR Emergency Committee concerning ongoing events and context involving transmission and international spread of poliovirus	Νo	Νo international travel or trade restrictions (5/5/2014 to 30/11/2018, ongoing)
SARS	27 March 2003/24 June 2003	Νο	WHO Scientific Research Advisory Committee SARS	Exit screening in affected countries (27/3 to 24/6/2003)	Νo international travel or trade restrictions (27/3/2003 to 24/6/2003)
Yellow fever	31 August 2016/16 May 2017	No	IHR Emergency Committee on yellow fever	No	Νo international travel or trade restrictions (31/8/2016 to 16/5/2017)
Zika virus disease	1 February 2016/18 November 2016	Yes (1/2/2016)	IHR Emergency Committee on Zika virus disease and observed increase in neurological disorders and neonatal malformations	No	Νo international travel or trade restrictions (1/2/2016 to 18/11/2016)

*Guinea, Liberia, and Sierra Leone. website: https://www.who.int/ihr/en/.

**Table 6:** Screening methods and accompanying measures.

Country/PoE/Type of Screening/Disease/Year(s)	Site of Assessment	Primary Screening			Secondary Screening			Accompanying Measures and Response Measures		
		Exposure Assessment	Symptom Assessment	Type of TMD *	Exposure Assessment	Symptom Assessment	Type of TMD	Accompanying Measures at the Border	Microbiological Tests	Quarantine/Isolation
Taiwan/airport/entry/Zika virus disease /2016 [7]	On board by crew or at aircraft site by airport officials	(-) *	Visual checks on board and at the terminal and temperature screening	Infrared cameras	Interview, questionnaire	In-person assessment	Ear thermometer	Risk communication (video), information about seeking medical assistance	Blood and urine samples sent to the Taiwan CDC’s laboratory (flaviviruses)	-
Taiwan/airport/entry/Dengue fever and Chikungunya infection/2013-2016 [7]	On board by crew or at airport site by airport officials	-	Visual checks on board and at the terminal and temperature screening	Infrared cameras	Interview, questionnaire	In-person assessment	Ear thermometer	Risk communication (video), information about seeking medical assistance	Dengue NS1 antigen rapid test (at airport), blood and urine samples sent to the Taiwan CDC’s laboratory (flaviviruses)	-
Taiwan/airport/entry/Dengue fever/2003-2007 [11]	Airport screening by airport clinicians	Questionnaire	Visual checks and temperature screening	Thermal scanning by non-contact infrared thermometers or infrared thermal camera	-	-	Ear thermometer	-	Real-time RT-PCR, and/or serological diagnosis by capture IgM/IgG ELISA	Not specified/at hospital
Taiwan/airport/entry/Dengue fever/2007-2010 [10]	Airport screening by quarantine officers	-	Questionnaire, temperature screening	Non-contact infrared thermometers (NCITs) with infrared thermal camera	Questionnaire	-	Ear thermometer	-	Dengue NS1 Rapid Test Kit (Bio-Rad, USA) (at airport), real-time RT-PCR, and/or serological diagnosis by capture IgM/IgG ELISA)	-
Australia/airport/entry/Ebola virus disease/2014-2015 [31]	At airport by public health staff and at public health units by infectious disease physician and public health unit staff	Questionnaire at terminal	Questionnaire at terminal	-	-	-	-	Risk communication, declaration of travel to EVD-affected countries on separate EVD-specific arrivals card, information about seeking medical assistance	PCR	At home/at designated Viral Hemorrhagic Fever hospital and local tertiary hospital
Belgium/airport/entry/Ebola virus disease/2014 [27]	-	-	Symptoms assessment (not specified) and temperature screening	Temperature screening (not specified)	-	-	-	Passenger locator card	In-house RT-PCR targets the GP gene; the large polymerase gene is targeted with an Altona commercial kit by Diagnostics GmbH, Hamburg, Germany	-
Japan/airport/entry/Ebola virus disease/2014-2015 [38]	At quarantine station, by immigration control officers	Interview	Questionnaire and temperature screening	Temperature screening (not specified)	-	-	-	Risk communication: posters at quarantine stations and in-flight announcements, information about seeking medical assistance	Laboratory test (not specified)	Not specified / hospital
UK/airport/entry/Ebola virus disease/2014-2015 [27,37]	At airport, by infectious disease physician	Questionnaire	Questionnaire and temperature screening	Ear thermometer	-	-	-	Information about seeking medical assistance	-	At home (asymptomatic but of high risk of EVD)/ At local acute hospital or designated specialist hospital
UK/train station/entry/Ebola virus disease/2014-2015 [27,37]	At train station, by infectious disease physician	Questionnaire	Questionnaire and temperature screening	Ear thermometer	-	-	-	Information about seeking medical assistance	-	Not specified/ At local acute hospital or designated specialist hospital
US/airport/entry/Ebola virus disease/2014-2015 [34]	At airport facilities	-	Temperature screening	Non-contact infrared thermometers	-	-	-	Risk communication, provision with Check and Report Ebola (CARE) kits that include health education materials, a thermometer, and ways to connect with their state or local health department, including a prepaid cell phone, information about seeking medical assistance	-	Unknown/Frontline health care facilities, Ebola assessment hospitals, and designated Ebola treatment centers
Guinea, Liberia, and Sierra Leone/airport/exit/Ebola virus disease/2014-2016 [6,16]	-	Questionnaire at terminal	Questionnaire at terminal, visual check, and temperature screening	Non-contact handheld infrared thermometer	Questionnaire at terminal	Questionnaire at terminal	Handheld non-contact thermometer	Risk communication, denied boarding	-	-
Guinea, Liberia, and Sierra Leone/land borders/exit /Ebola virus disease/2014-2016 [16]	-	-	Visual checks	-	-	-	-	-	-	-
Guinea, Liberia, and Sierra Leone/seaport/exit/Ebola virus disease/2014-2016 [16]	-	-	Temperature screening	-	-	-	-	Restricted access to vessels in port and disembarkation of seafarers, including cancellation of shore passes and crew transfers Protective equipment requirements for staff required to board vessels	-	Established on-site isolation facilities
China/airport/entry/Influenza Pandemic (H1N1) 2009 [40]	At quarantine station of the airport	-	Visual checks on board and temperature screening on board	-	Questionnaire at terminal	Questionnaire at terminal	-	Information about seeking medical assistance	RT- PCR	Not specified/ At the community hospitals or quarantine station, by general practitioners or public health workers
Japan/airport/entry/Influenza Pandemic (H1N1) 2009^#^ [39]	On board, at terminal	Questionnaire	Questionnaire and temperature screening	Handheld infrared thermoscanner and axillary or oral on board, a fixed infrared thermoscanner at terminal	Questionnaire	Questionnaire	Ear or axillary thermometer	Information about seeking medical assistance, entry card	Rapid influenza test (on board), RT-PCR	Close contacts were quarantined at designated hotels/at designated medical institution
Japan/airport/entry/ Influenza Pandemic (H1N1) 2009 ^‡^ [39]	On board, at terminal	Questionnaire	Questionnaire and temperature screening	Handheld infrared thermoscanner on board, a fixed infrared thermoscanner at terminal	Questionnaire	Questionnaire	-	Information about seeking medical assistance, entry card	Rapid influenza test (on board and at terminal), RT-PCR	At designated medical institution
New Zealand/airport/entry/Influenza Pandemic (H1N1) 2009 [21]	-	-	Visual checks	-	-	-	-	Risk communication (in-flight scripted health message, signs), locator card completion	RT-PCR	At home or a facility for isolation
Peru/airport/entry/Influenza Pandemic (H1N1) 2009 [32]	-		Temperature screening	-	-	-	-		RT-PCR	
Singapore/airport/entry/Influenza Pandemic (H1N1) 2009 [36]	-	-	Temperature screening	Thermal scanners	-	-	-	Risk communication (health advisories), information about seeking medical assistance	RT-PCR	At designated screening center at Hospital
Australia /airport/entry/Influenza Pandemic (H1N1) 2009 [20]	At terminal	Health declaration card	Health declaration card	Non-contact thermal imaging scanners	-	Brief clinical assessment	-	-	-	-
Taiwan/airport/entry/Influenza Pandemic (H1N1) 2009 [33]	-	-	Temperature screening	Infrared fever cameras	-	-	-	Health protection materials, such as masks and gloves, risk communication (broadcasting voice recording and distributing education sheets), information about seeking medical assistance	Laboratory test (not specified)	Not specified/At contract hospitals
Australia/airport/entry/SARS/2003 [8]	On board/at terminal	-	-	-	-	In-person assessment	Ear thermometer	Risk communication (in-flight notification by airline staff)	-	At home, by nurse and Chief Quarantine Officer/At designated state or territory hospital, by nurse and Chief Quarantine Officer
Australia/seaport/entry/SARS/2003 [8]	On board/at terminal	-	-	-	-	In-person assessment	Ear thermometer	Risk communication	-	
Canada/airport/entry/ exit/SARS/2003 [9]	-	Questionnaire (health alert notice)	Questionnaire (health alert notice) and temperature screening	Thermal scanning machines	Questionnaire	Questionnaire	Oral thermometer	Risk communication (posters and health alert notices) information about seeking medical assistance, traveler contact information form	PCR, serological test	Unknown/ At a predetermined hospital
Singapore/airport, seaport, road entry points/entry/SARS/2003 [23,29]	-	Health declaration cards	Visual checks and temperature screening	Thermal scanners	-	-	-	Risk communication, information about seeking medical assistance	Serology, SARS antibodies, and/or SARS coronavirus PCR	Home/ At specific hospital
Taiwan/airport/entry/SARS/2003 [35]	-	-	Interview, questionnaire	Infrared cameras	-	-	Ear electronic thermometer	Risk communication (public media), information about seeking medical assistance	White blood cell count, Chest X-ray findings	At specific hospital

TMD: Temperature measuring device, CDC: Center for Disease Control and Prevention, RT-PCR: Reverse Transcription Polymerase Chain Reaction, SARS: Severe Acute Respiratory Syndrome. * (-) Not specified, ^#^ from 28 April to 21 May 2009, ^‡^ from 22 May to 18 June 2009.

**Table 7:** Number of travelers screened, suspected and confirmed cases detected through entry screening.

Targeting Disease/s	Country	Timeframe	Number of Travelers			Sensitivity	Specificity	Country Surveillance Data/ Imported Cases **	Reference
			Screened	Suspected	Confirmed Detected				
SARS	Taiwan	3 March to 22 April 2003	2819	46	0	-	-	346/unknown	[35]
	Canada	14 May to 5 July 2003	For health alert notices inbound: 677,494; For thermal imaging scanner inbound: 467,870	For health alert notices inbound: 2478; For thermal imaging scanner inbound: 95	0	-	-	251/unknown	[9]
	Australia	5 April to 16 June 2003	1.84 million arrivals	794 were referred for screening to quarantine and inspection service staff. Of these, 734 (92.4%) were referred by quarantine/inspection service staff to the nurses at airports. 19 (2.4%) were then referred to the Chief Quarantine Officer	0	-	-	6/unknown	[8]
	Singapore	31 March to 31 May 2003	442,973	136	0	-	-	238/unknown	[23]
	Singapore	9 April to 21 Sept 2003	No information available	4044 travelers were detected to have temperatures >37.5 °C through screening at the airport and sea terminals. Of these travelers, 327 were referred to hospital for assessment and 39 were admitted for further evaluation and isolation.	0	-	-	238/unknown	[29]
Influenza Pandemic (H1N1) 2009	Singapore	27 April to 27 June 2009	-	-	14	-	-		[36]
	Australia	28 April to 18 June 2009	625,147	5845 (0.93%) identified as symptomatic or febrile, 1296 (22.17%) identified as requiring further clinical assessment	3	6.67% (95% CI, 1.40–18.27%) *	99.10% (95% CI, 99.00–100.00%) *		[20]
	Japan	28 April to 20 June	120,069	391 cases (0.33%)	5 (1.28%) influenza type A 1 as influenza type B	-	-		[26]
	Japan	28 April to 18 June 2009	441,041 passengers and 30,692 airline crew members	805	15	-	-		[24]
	Japan	1 September 2009 to 31 January 2010	9,140,435	1049	10	-	-		[24]
	Japan	Period I: 28 April to 21 May 2009, Period II: 22 May to 18 June 2009	Period I: 20,603; Period II: 265,696	Period I: 561, Period II: 244	Period I: 4, Period II: 5	-	-		[39]
	Taiwan	From 27 April to 19 June 2009	1,732,455	2685 were detected ^£^ to have suspicious symptoms, including 1303 fever cases. Among these fever cases, 184 were sent to hospitals for further diagnosis and treatment after they were evaluated in terms of travel history and symptoms, by quarantine physicians or quarantine officers.	12				[33]
	China	16 May to 30 September 2009	600,000		132	-	-		[40]
	Peru	24 April to 4 July 2009	500,000	0	0	-	-		[32]
	New Zealand	27 April to 22 June 2009	456,518	406 (0.09%) of these were referred for medical assessment. Of those, 109 (27%) met the case definition and received virologic testing.	4	5.8% (95% CI 2.3–14.0%) ^†^	-		[21]
Ebola virus disease	US		No information available	>1200 travelers were referred to CDC for additional screening because of illness or, more commonly, to assess possible exposures; 28 persons were referred for medical evaluation.	0	-	-	-	[28]
	US	11 October to 10 November 2014	1993	86 (4.3%)	0	-	-	-	[6]
	US	11 October 2014 to 31 January 2015	7587	543 (7.2%) were referred to on-site CDC screening at the airport for additional exposure risk assessment. At the time of assessment, 12 (0.16%) travelers were referred for medical evaluation at a local hospital.	0	-	-	11/9	[34]
	Australia	1 October 2014 to 13 April 2015	123	6	0	-	-	-	[31]
	Belgium	20 October 2014 to 17 March 2015	13,356	0	0	-	-	-	[27]
	UK	October 2014 to March 2015	Approximately 5000	9	0	-	-	1/1	[27]
	UK	14 October 2014 to 4 January 2015	3388 passengers screened at UK ports of entry	125 low risk passengers 5 high-risk passengers	0	-	-	1/1	[37]
	Japan	August 2014 to September 2015	9	9	0	-	-	-	[38]
Zika virus disease	Taiwan	January to October 2016	21,083,404	21,721 were identified as potentially ill through fever screening or passengers’ self-reporting. Upon evaluation, 3199 specimens were collected.	5		-	17/17	[7]
Dengue fever	Taiwan	2007 to 2010	52,047,769	48,115	406	40.22% (2007), 44.44% (2008), 53.2% (2009), 41.86% (2010) *	99.96% (2007), 99.96% (2008), 99.97% (2009), 99.97% (2010) *	5,800/910	[10]
	Taiwan	2013 to 2016	85,464,274	67,704 ill passengers detected by entry screening, 9944 specimens collected	518	-	-	61,118/1,249	[7]
	Taiwan	2003 to 2007				-	-	4119/539	[11]
	Taiwan	July 2003 to June 2004	8,000,000	≈22,000 passengers were identified as fever patients. After clinical diagnosis, 3011 serum samples were sent for laboratory diagnosis of Dengue virus infection.	40	-	-	6005/73	[15]
Chikungunya infection	Taiwan	2013 to 2016	85,464,274	67,704 ill passengers detected by entry screening, 9944 specimens collected	29	-	-	91/48	[7]

* Visual and fever screening, medical and laboratory assessment and questionnaire were applied. ^†^ Visual screening, medical and laboratory assessment were applied. ^£^ Visual and fever screening, medical and laboratory assessment were applied. ** Data in this column were extracted from papers and websites other than those fulfilling the eligibility criteria, which are listed in the last column of the table [15,41,42,43,44,45,46,47,48].

**Table 8:** Number of travelers screened, suspected and confirmed cases identified through exit screening.

Country	Targeting Disease/s	Timeframe	Number of Travelers				Sensitivity	Specificity	Reference
			Screened	Suspected	Confirmed Detected	Confirmed Not Detected			
Guinea, Liberia, Sierra Leone	Ebola virus disease	August to October 2014	80,000		0	No	-	-	[6]
Canada	SARS	14 May to 5 July 2003	For health alert notices: 495,492 For thermal imaging scanner: 295,212	For health alert notices: 411 For thermal imaging scanner: 96	0	No	-	-	[9]
Guinea, Liberia, Sierra Leone, Nigeria, Senegal, and Mali	Ebola virus disease		>200,000 travelers leaving Guinea, Liberia, and Sierra Leone had been screened and >150,000 in Nigeria				-	-	[28]
Guinea, Liberia, and Sierra Leone	Ebola virus disease	12 August to 12 October 2014	36,000	77	0	1	0% *	99.79% *	[49]
Guinea, Liberia, and Sierra Leone	Ebola virus disease	August 2014 to January 2016	300,000		0	4 (none of them was overtly symptomatic at the time of travel)	-	-	[16]

SARS: Severe Acute Respiratory Syndrome. * Visual and fever screening, medical and laboratory assessment, and questionnaire were applied.

**Table 9:** Reported facts about the cost of screening measure.

Disease	Type of Screening and Setting	Cost of Measures	Recommendations	Reference
Severe Acute Respiratory Syndrome	General, ad hoc entry/exit screening at airport in Canada	*An estimated Can$ 7.55 million was invested in airport screening measures from March 18 to July 5, 2003.*	*“Rather than investing in airport screening measures to detect rare infectious diseases, investments should be used to strengthen screening and infection control capacities at points of entry into the healthcare system.”*	[9]
Influenza Pandemic (H1N1) 2009	General, ad hoc entry screening at airport in New South Wales (NSW), Australia	*The cost of staffing airport clinics in NSW has been estimated at about US$50,000 per case detected (NSW Ministry of Health, unpublished data, 2012).*	*“Given the costs associated with staffing airport clinics, careful consideration should be given to deploying resources to airports for largely ineffective screening measures, compared with other activities such as contact tracing in the community”.*	[20]
Dengue fever	General, entry screening at airport on routine basis in Taiwan	*Not addressed.*	*“Our evaluation of the routine border screening for Dengue using NCITs yielded a low Positive Predictive Value, which suggested a low cost-effectiveness”.*	[10]
		*The airport fever screening method requires an infrared thermal camera, which costs approximately US$ 43,000 for each set of instruments. In addition, one additional worker is needed to monitor this alarm system.*	*“The cost of identifying Dengue virus infections with airport fever screening is similar to that of other surveillance methods. The porting procedure and clinical and laboratory diagnoses are similar to those of surveillance methods. Therefore, the method is a cost-effective means of identifying imported Dengue cases”.*	[15]

**Table 10:** Assessment of public health impact as reported by authors.

Disease/Type of Screening/ Point of Entry/Country	Methods	Results	References
Dengue fever/ Entry screening on routine basis/ Airport/Taiwan	Comparing confirmed cases identified at points of entry with total imported cases	*“Airport fever screening was successful in identifying 45% (244/542; 95% confidence interval 33.1–57.8%) of imported Dengue cases with fever.”*	[11]
Dengue fever/ Entry screening on routine basis/ Airport/Taiwan	Fluctuations in the number of symptomatic imported Dengue cases identified in the airports (X) were associated with the total number of imported Dengue cases (Y) based on a regression analysis of a biweekly surveillance	*“By implementing the airport fever screening program followed by laboratory confirmation, nearly half of the imported symptomatic Dengue cases were detected at entry.”* *“An analysis of the dataset according to the geographical areas (25 counties/cities) indicated that there were significant correlations between the annual cumulative number of Dengue importations identified at the airports (X) and the number of Dengue importations reported from community clinics (Y) (n = 96, Y = 0.93X + 1.208, R2 = 0.57, p < 0.0001).”*	[10]
Dengue fever/ Entry screening on routine basis/ Airport/Taiwan	Comparing confirmed cases identified at points of entry with total imported cases	*“518/1188 confirmed cases identified at points of entry/total imported cases (43.6%)”*	[7]
Dengue fever/ Entry screening on routine basis/ Airport/Taiwan	Comparing confirmed cases identified at points of entry with total imported cases. Comparing numbers of imported cases before and after screening measure implementation	*“Airport fever screening alone identified 40 (83.3%) of 48 of all imported cases identified by the active surveillance system.”* *“Fever screening at the airports has also dramatically increased the proportion of imported Dengue cases identified by active surveillance, 48 (65.8%), of 73 which is significantly higher than the number identified during years before fever screening were implemented (p < 0.0001 by chi-square test)”*	[15]
Influenza Pandemic (H1N1) 2009/ Entry screening ad hoc/ Airport/Japan	Comparing cases in the community and imported cases identified through screening	*“In spite of the quarantine inspection, the number of Japanese patients with novel influenza reached 633 by June 18, 2009. Only 11 patients were found by the airport quarantine inspection, but importantly, about 20% of all patients had an overseas travel history and had passed through the quarantine inspection.”*	[26]
Influenza Pandemic (H1N1) 2009/ Entry screening ad hoc/ Airport/Japan	Comparing surveillance data of imported cases with entry screening results and investigating imported cases’ travel history and time of symptoms onset	*“6.6% (10/151) of the individuals infected during international travel were identified by the border control measures upon entry in May and June 2009.”* *“2 individuals among those identified later in Japan to be infected had been missed at entry despite being symptomatic. 22 others were identified after entry into Japan despite being symptomatic at entry screening.”* *“Health monitoring identified 8 infected individuals. Enhanced surveillance identified 812 individuals, 141 (18%) of whom had a history of international travel. 24 these 141 passengers picked up by enhanced surveillance had been developing symptoms on entry and were missed at screening.”*	[39]
Influenza Pandemic (H1N1) 2009/ Entry screening ad hoc/ Airport/Taiwan	Comparing surveillance data of imported cases with entry screening results	*“Cases identified among passengers screened out by quarantine measures and transferred to hospitals by quarantine officers account for 20.3% (12 cases) of all imported cases.”*	[33]
Zika virus disease/ Entry screening ad hoc/ Airport/ Taiwan	Comparing confirmed cases identified at points of entry with total imported cases	*“As of October 31, 2016, Taiwan has no locally acquired Zika infections, but 13 imported cases have been identified, of which 38% were identified by airport border screening.”*	[7]
Chikungunya infection / Entry screening ad hoc/ Airport/ Taiwan	Comparing confirmed cases identified at points of entry with total imported cases	*“29/48 Confirmed cases identified at points of entry/total imported cases (60.4%)”*	[7]

**Figure A1:**
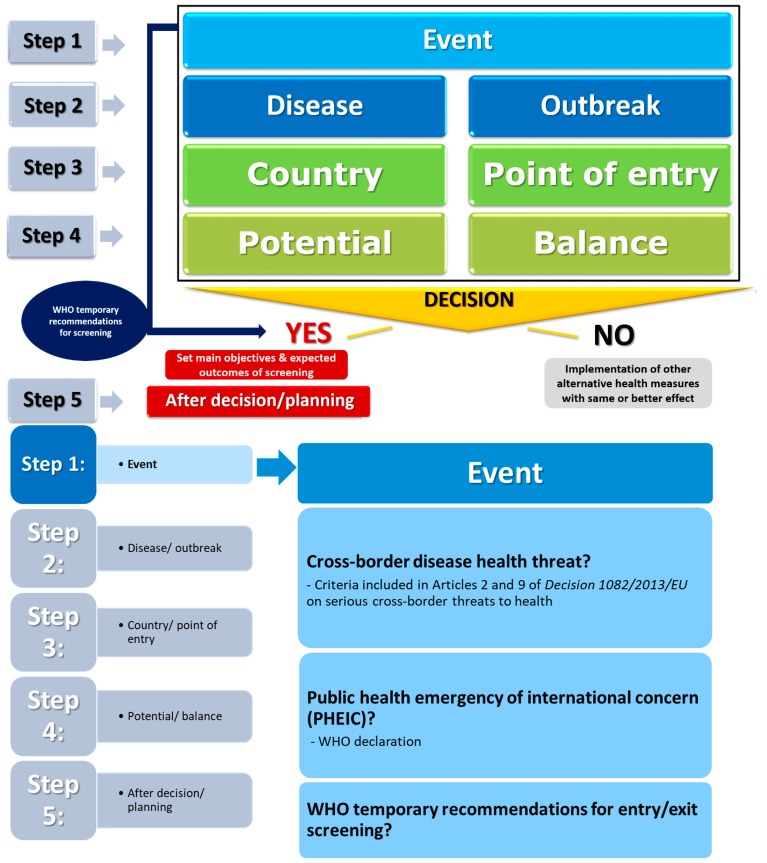

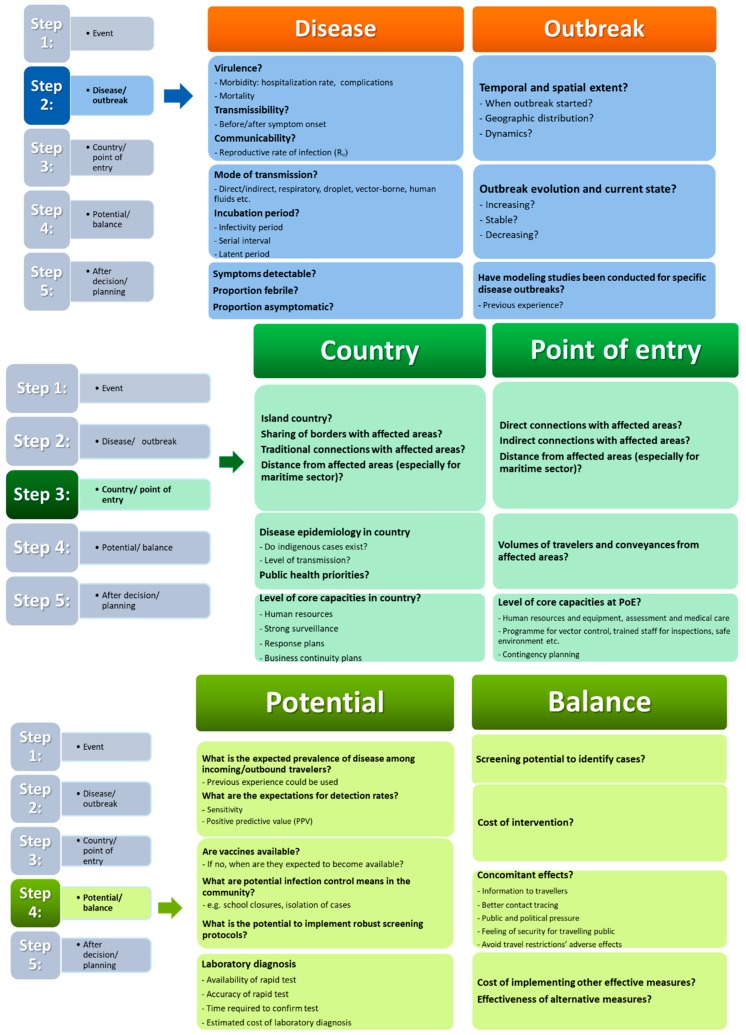

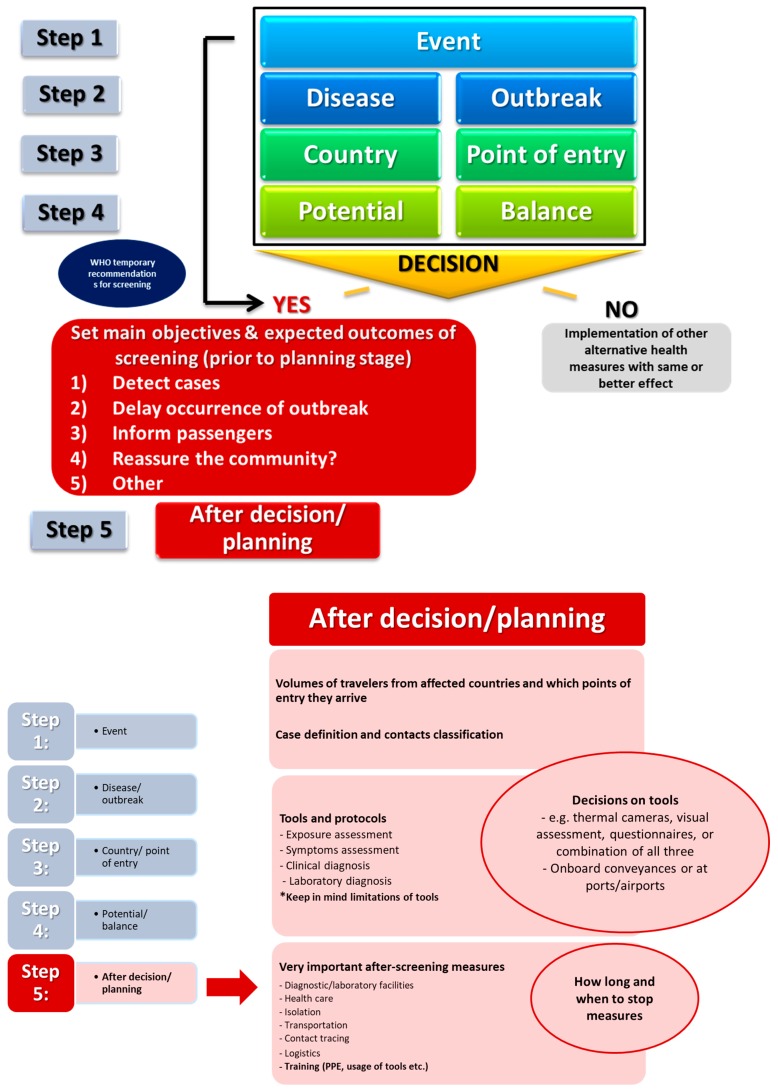
Algorithm for making evidence-based decisions in implementing entry and exit screening measures at points of entry.

## Appendix A

**Table A1:** List of websites searched as part of the grey literature.

Country/Organization	Website
WHO	https://who.int/; http://www.who.int/about/regions/afro/en/; http://www.who.int/about/regions/amro/en/; http://www.who.int/about/regions/searo/en/; http://www.who.int/about/regions/euro/en/; http://www.who.int/about/regions/emro/en/; http://www.who.int/about/regions/wpro/en/
ECDC	https://ecdc.europa.eu
Austria	https://www.bmgf.gv.at
Belgium	https://www.sciensano.be
Bulgaria	https://www.ncipd.org
Croatia	https://www.hzjz.hr
Republic of Cyprus	https://www.moh.gov.cy/moh/
Czech Republic	http://www.szu.cz
Denmark	https://www.sst.dk
Estonia	http://www.terviseamet.ee
Finland	https://thl.fi
France	http://www.santepubliquefrance.fr
Germany	https://www.rki.de
Greece	http://www.keelpno.gr
Hungary	http://www.kormany.hu
Ireland	http://www.hpsc.ie
Italy	http://www.salute.gov.it
Latvia	https://www.spkc.gov.lv
Lithuania	http://sam.lrv.lt
Luxembourg	http://www.sante.public.lu
Malta	https://deputyprimeminister.gov.mt
Netherlands	https://www.rivm.nl
Poland	http://www.pzh.gov.pl
Portugal	https://www.dgs.pt
Romania	http://www.insp.gov.ro
Slovakia	http://www.uvzsr.sk
Slovenia	http://www.nijz.si
Spain	http://www.msssi.es
Sweden	http://www.smittskyddsinstitutet.se
the UK	https://www.gov.uk/government/organisations/public-health-england
Iceland	https://www.landlaeknir.is
Liechtenstein	https://www.llv.li
Norway	https://www.fhi.no
Switzerland	https://www.swisstph.ch
US CDC	https://www.cdc.gov/
Taiwan	https://www.cdc.gov.tw
China	http://www.chinacdc.cn/en/
Hong Kong	https://www.chp.gov.hk
India	https://phfi.org
Brazil	https://portal.fiocruz.br
Canada	http://www.ciphi.ca
Nigeria	https://ncdc.gov.ng
Liberia	http://moh.gov.lr/, http://liberiamohsw.org/Policies&Plan.html
Sierra Leone	http://health.gov.sl, http://gov.sl/ministry-health-and-sanitation
Mali	http://www.sante.gov.ml/
ICAO	https://www.icao.int
IATA	https://www.iata.org
CAPSCA	https://www.capsca.org
ACI	https://aci.aero/
CLIA	https://cruising.org
ISF	http://www.allaboutshipping.co.uk
UIC	https://uic.org
OTIF	https://otif.org
OSJD	http://en.osjd.org
CIT	https://www.cit-rail.org
ERRAC	http://errac.org

## Appendix B. Questionnaire for Checking on Eligibility Criteria

Title of article: ……………………………………………………………..………………………… Author: …………………………………… Year of publication: …………………

a. Type of screening (entry, exit) **Yes** **☐ No** **☐**
b. Types of infectious disease or diseases that entry and exit screening was targeting **Yes** **☐ No** **☐**
c. Type of points of entry (airports, ports, ground crossings) where measures were implemented **Yes** **☐ No** **☐**
d. Screening carried out on a routine basis or on an ad hoc basis after a public health event has occurred and its purpose **Yes** **☐ No** **☐**
e. Description of methods used in entry/exit screening (primary/secondary, questionnaire, body temperature check, technology used) **Yes** **☐ No** **☐**
f. Type of technology used (thermometers, scan cameras, or other technology)
g. After screening, the applied diagnosis protocol (laboratory and clinical examination)
h. Number of cases identified for a specific timeframe **Yes** **☐ No** **☐**
i. Total numbers of travelers screened for a specific timeframe **Yes** **☐ No** **☐**
j. Percentage of persons positive to screening that were diagnosed with the targeted disease **Yes** **☐ No** **☐**
k. Percentage of persons diagnosed with different disease from the initially targeted for the specific timeframe (positive and negative predictive values) **Yes** **☐ No** **☐**
l. General screening or targeted screening (e.g., traveler from any affected outbound country, all travelers directly arriving only from affected countries/areas, nationality of travelers, travelers in-transit that have called an affected country/areas (West Africa)) **Yes** **☐ No** **☐**
m. Reporting on the assessment of the public health impact of the entry/exit screening measures or the cost-effectiveness of methods applied

## Appendix C. Questionnaire for Data Extraction

- Type of screening: entry ☐ exit ☐
- Types of infectious disease or diseases that entry and exit screening was targeting
- Type of points of entry: airports ☐ ports ☐ ground crossings ☐
- Screening carried out on a routine basis ☐ or on an ad hoc basis ☐ after a public health event has occurred
- Methods used in entry/exit screening: primary ☐ secondary ☐ questionnaire ☐ body temperature ☐ technology used ☐ else
- Type of technology used: thermometers scan cameras
- After screening, the applied diagnosis protocol (laboratory and clinical examination)
- Number of cases identified and the total numbers of travelers screened
- Percentage of persons positive to screening finally diagnosed
- Percentage of persons diagnosed with different disease from the initially targeted
- The applied protocol after diagnosis and management of cases
- Health measures applied to the traveler and the environment
- General screening or targeted screening: outbound country ☐ travelers directly arriving from affected countries ☐ nationality ☐ travelers in-transit ☐
- Inter-sectorial collaboration and coordination processes
- Involved officers: Public health officers ☐ ministry officers ☐ regional health system ☐ national health system ☐ NGOs ☐ else ☐
- Concrete example of entry/exit screening
- Practices, experiences, and lessons learnt reported
- Challenges reported (limitations, failures, mishaps)
- Bad practices reported
- Methods used to assess the public health impact of the entry/exit screening and their result
- Methods used to appraise the cost-effectiveness of screening method and results
- Evaluation of method results: sensitivity ☐ specificity ☐ false positive/negative ☐ (of screening method) ☐ positive and negative predictive values
- Decision-making level: Public health officers ☐ ministry officers ☐ regional ☐ national ☐ intersectoral collaboration ☐ health and border authorities ☐
- Communication channels
- Notification practices between neighboring and possibly affected countries
- Specific timeframe referred Yes ☐ No ☐ Duration:

## Appendix D. List of Publications Identified through the Scoping Search

**Assessment for imported cases notification of infectious diseases**

CHIEN-HUA CHU, J. H., YA-LING CHEN, CHE-CHIEH YEN, 2011. Study on the Notification of Imported Cases of Notifiable Diseases by Using the Data in the National Health Insurance Information System during 2007–2008. Taiwan Epidemiology Bulletin, 27, 19–38.

**Dengue entry screening at airports**

KUAN, M. M. & CHANG, F. Y. 2012. Airport sentinel surveillance and entry quarantine for Dengue infections following a fever screening program in Taiwan. BMC Infect Dis, 12, 182.

SHU, P. Y., CHIEN, L. J., CHANG, S. F., SU, C. L., KUO, Y. C., LIAO, T. L., HO, M. S., LIN, T. H. & HUANG, J. H. 2005. Fever screening at airports and imported Dengue. Emerging Infectious Diseases, 11, 460–462.

**Ebola Virus Disease**

**Preparedness and response planning for Ebola Virus Disease**

BROSH-NISSIMOV, T., POLES, L., KASSIRER, M., SINGER, R., KALINER, E., SHRIKI, D. D., ANIS, E., FOGEL, I., ENGELHARD, D., GROTTO, I. & ISRAELI EPIDEMIC MANAGEMENT, T. 2015. Preparing for imported Ebola cases in Israel, 2014 to 2015. Euro Surveill, 20.

DEMOCRATIC REPUBLIC OF THE CONGO 2018. Strategic response plan for the Ebola virus disease outbreak.

ECDC 2014. Technical report: Infection prevention and control measures for Ebola virus disease Entry and exit screening measures.

ECDC 2015. Mission Report: Public health emergency preparedness for cases of viral hemorrhagic fever (Ebola) in Belgium: a peer review-16–19 March 2015.

FRIEDEN, T. R. & DAMON, I. K. 2015. Ebola in West Africa—CDC’s Role in Epidemic Detection, Control, and Prevention. Emerg Infect Dis, 21, 1897-905.

NUNN, R., JAWAD, M., CRUICKSHANK, H., POOLE, R., VASS, C. & FRASER, S. 2015. Perspectives on Ebola screening at ports of entry in the UK. Perspect Public Health, 135, 66–67.

WORLD HEALTH ORGANISATION 2014a. Exit Screening Ebola Guidance Package.

WORLD HEALTH ORGANISATION 2014b. Technical note for Ebola virus disease preparedness planning for entry screening at airports, ports, and land crossings.

WORLD HEALTH ORGANISATION 2014c. WHO Interim Guidance for Ebola Virus Disease: Exit Screening at Airports, Ports, and Land Crossings.

WORLD HEALTH ORGANISATION 2014d. WHO Interim Guidance for Ebola Event Management at Points of Entry.

WORLD HEALTH ORGANISATION 2018d. WHO Regional Strategic EVD Readiness Preparedness Plan.

**Entry/exit screening measures for Ebola Virus Disease experience**

BROWN, C. M., ARANAS, A. E., BENENSON, G. A., BRUNETTE, G., CETRON, M., CHEN, T. H., COHEN, N. J., DIAZ, P., HABER, Y., HALE, C. R., HOLTON, K., KOHL, K., LE, A. W., PALUMBO, G. J., PEARSON, K., PHARES, C. R., ALVARADO-RAMY, F., ROOHI, S., ROTZ, L. D., TAPPERO, J., WASHBURN, F. M., WATKINS, J., PESIK, N., CENTERS FOR DISEASE, C. & PREVENTION 2014. Airport exit and entry screening for Ebola--August-November 10, 2014. MMWR Morb Mortal Wkly Rep, 63, 1163–1167.

CARDILE, A. P., MURRAY, C. K., LITTELL, C. T., SHAH, N. J., FANDRE, M. N., DRINKWATER, D. C., MARKELZ, B. P. & VENTO, T. J. 2015. Monitoring Exposure to Ebola and Health of US Military Personnel Deployed in Support of Ebola Control Efforts - Liberia, October 25, 2014–February 27, 2015. MMWR Morb Mortal Wkly Rep, 64, 690–694.

CDC 2015. Interim Table of State Ebola Screening and Monitoring Policies for Asymptomatic Individuals.

CHAN, J., PATEL, M., TOBIN, S. & SHEPPEARD, V. 2017. Monitoring travelers from Ebola-affected countries in New South Wales, Australia: what is the impact on travelers? BMC Public Health, 17, 113.

COHEN, N. J., BROWN, C. M., ALVARADO-RAMY, F., BAIR-BRAKE, H., BENENSON, G. A., CHEN, T. H., DEMMA, A. J., HOLTON, N. K., KOHL, K. S., LEE, A. W., MCADAM, D., PESIK, N., ROOHI, S., SMITH, C. L., WATERMAN, S. H. & CETRON, M. S. 2016. Travel and Border Health Measures to Prevent the International Spread of Ebola. MMWR Suppl, 65, 57–67.

CROOK, P., SMITH-PALMER, A., MAGUIRE, H., MCCARTHY, N., KIRKBRIDE, H., COURT, B., KANAGARAJAH, S., TURBITT, D., AHMED, S., COSFORD, P. & OLIVER, I. 2017. Lack of Secondary Transmission of Ebola Virus from Healthcare Worker to 238 Contacts, United Kingdom, December 2014. Emerg Infect Dis, 23, 2081–2084.

GILBERT, G. L. 2016. Australia’s response to Ebola Virus disease in West Africa, 2014–2015. Public Health Res Pract, 26.

GULLAND, A. 2014. Experts question usefulness of screening travelers to UK for Ebola. BMJ, 349, g6199.

JACOBSON, S. H., YU, G. & JOKELA, J. A. 2016. A double-risk monitoring and movement restriction policy for Ebola entry screening at airports in the United States. Prev Med, 88, 33–38.

KALRA, S., KELKAR, D., GALWANKAR, S. C., PAPADIMOS, T. J., STAWICKI, S. P., ARQUILLA, B., HOEY, B. A., SHARPE, R. P., SABOL, D. & JAHRE, J. A. 2014. The emergence of Ebola as a global health security threat: from ‘lessons learnt’ to coordinated multilateral containment efforts. J Glob Infect Dis, 6, 164–177.

KEITA, M., CONTE, F., DIALLO, B., LUFWA, D., KATOMBA, J., SNACKEN, R., PALLAWO, R., TOLNO, A., DIALLO, A. B., DJINGAREY, M. H. & SUBISSI, L. 2017. Lessons learnt by surveillance during the tail-end of the Ebola outbreak in Guinea, June-October 2015: a case series. BMC Infect Dis, 17, 304.

KESTEN, J. M., AUDREY, S., HOLDING, M., COOPE, C., YOUNG, N., BROWN, C. S., HARRIES, J., HICKMAN, M. & OLIVER, I. 2018. Qualitative study of Ebola screening at ports of entry to the UK. BMJ Glob Health, 3, e000788.

C., HARVEY, M. C., PIETZ, H., BERTOLLI, J., PERZ, J. F., WHITNEY, C. G., HALPIN, A. S., DALEY, W. R., PESIK, N., MARGOLIS, G. S., TUMPEY, A., TAPPERO, J., DAMON, I., CENTERS FOR DISEASE, C. & PREVENTION 2015. Systems for rapidly detecting and treating persons with ebola virus disease--United States. MMWR Morb Mortal Wkly Rep, 64, 222–225.

KOONIN, L. M., JAMIESON, D. J., JERNIGAN, J. A., VAN BENEDEN, C. A., KOSMOS,

KRAEMER, J. D., SIEDNER, M. J. & STOTO, M. A. 2015. Analyzing Variability in Ebola-Related Controls Applied to Returned Travelers in the United States. Health Secur, 13, 295–306.

MCCARTHY, M. 2014. US increases Ebola screening at five airports. BMJ, 349, g6147.

MILLMAN, A. J., CHAMANY, S., GUTHARTZ, S., THIHALOLIPAVAN, S., PORTER, M., SCHROEDER, A., VORA, N. M., VARMA, J. K. & STARR, D. 2016. Active Monitoring of Travelers Arriving from Ebola-Affected Countries - New York City, October 2014–April 2015. MMWR Morb Mortal Wkly Rep, 65, 51–54.

MIRKOVIC, K., THWING, J., DIACK, P. A., CENTERS FOR DISEASE, C. & PREVENTION 2014. Importation and containment of Ebola virus disease - Senegal, August-September 2014. MMWR Morb Mortal Wkly Rep, 63, 873–874.

MOLL, R., REECE, S., COSFORD, P. & KESSEL, A. 2016. The Ebola epidemic and public health response. Br Med Bull, 117, 15–23.

NYENSWAH, T. G., KATEH, F., BAWO, L., MASSAQUOI, M., GBANYAN, M., FALLAH, M., NAGBE, T. K., KARSOR, K. K., WESSEH, C. S., SIEH, S., GASASIRA, A., GRAAFF, P., HENSLEY, L., ROSLING, H., LO, T., PILLAI, S. K., GUPTA, N., MONTGOMERY, J. M., RANSOM, R. L., WILLIAMS, D., LANEY, A. S., LINDBLADE, K. A., SLUTSKER, L., TELFER, J. L., CHRISTIE, A., MAHONEY, F. & DE COCK, K. M. 2016. Ebola and Its Control in Liberia, 2014-2015. Emerg Infect Dis, 22, 169–177.

PARHAM, M., EDISON, L., SOETEBIER, K., FELDPAUSCH, A., KUNKES, A., SMITH, W., GUFFEY, T., FETHEROLF, R., SANLIS, K., GABEL, J., COWELL, A., DRENZEK, C., CENTERS FOR DISEASE, C. & PREVENTION 2015. Ebola active monitoring system for travelers returning from West Africa-Georgia, 2014-2015. MMWR Morb Mortal Wkly Rep, 64, 347–350.

PUBLIC HEALTH AGENCY OF CANADA 2014. Statement on infection prevention and control measures for Ebola virus disease (December 2014). Can Nurse, 110, 17-8.

READ, J. M., DIGGLE, P. J., CHIROMBO, J., SOLOMON, T. & BAYLIS, M. 2015. Effectiveness of screening for Ebola at airports. Lancet, 385, 23-24.

SAITO, T. 2015. Public health challenges and legacies of Japan’s response to the Ebola virus disease outbreak in West Africa 2014 to 2015. Eurosurveillance, 20, 25–30.

SHUAIB, F., GUNNALA, R., MUSA, E. O., MAHONEY, F. J., OGUNTIMEHIN, O., NGUKU, P. M., NYANTI, S. B., KNIGHT, N., GWARZO, N. S., IDIGBE, O., NASIDI, A., VERTEFEUILLE, J. F., CENTERS FOR DISEASE, C. & PREVENTION 2014. Ebola virus disease outbreak—Nigeria, July–September 2014. MMWR Morb Mortal Wkly Rep, 63, 867–872.

STEHLING-ARIZA, T., FISHER, E., VAGI, S., FECHTER-LEGGETT, E., PRUDENT, N., DOTT, M., DALEY, R. & AVCHEN, R. N. 2015. Monitoring of Persons with Risk for Exposure to Ebola Virus Disease - United States, November 3, 2014-March 8, 2015. MMWR Morb Mortal Wkly Rep, 64, 685–689.

SUNSHINE, G., PEPIN, D., CETRON, M. & PENN, M. 2015. State and Territorial Ebola Screening, Monitoring, and Movement Policy Statements - United States, August 31, 2015. MMWR Morb Mortal Wkly Rep, 64, 1145–1146.

WHO-ICAO 2014. Joint Statement on EVD outbreak.

**Studies about evolution and predictions of Ebola Virus Disease spread**

ARWADY, M. A., BAWO, L., HUNTER, J. C., MASSAQUOI, M., MATANOCK, A., DAHN, B., AYSCUE, P., NYENSWAH, T., FORRESTER, J. D., HENSLEY, L. E., MONROE, B., SCHOEPP, R. J., CHEN, T. H., SCHAECHER, K. E., GEORGE, T., ROUSE, E., SCHAFER, I. J., PILLAI, S. K. & DE COCK, K. M. 2015. Evolution of ebola virus disease from exotic infection to global health priority, Liberia, mid-2014. Emerg Infect Dis, 21, 578–584.

BOGOCH, II, CREATORE, M. I., CETRON, M. S., BROWNSTEIN, J. S., PESIK, N., MINIOTA, J., TAM, T., HU, W., NICOLUCCI, A., AHMED, S., YOON, J. W., BERRY, I., HAY, S. I., ANEMA, A., TATEM, A. J., MACFADDEN, D., GERMAN, M. & KHAN, K. 2015. Assessment of the potential for international dissemination of Ebola virus via commercial air travel during the 2014 west African outbreak. Lancet, 385, 29–35.

INTERNATIONAL CIVIL AVIATION ORGANISATION 2014. Recognizing the role of aviation in the Ebola outbreak and other public health emergencies. 51st Conference of Directors General of Civil Aviation Asia and pacific Regions.

**Entry/exit screening measures for infectious diseases**

BITAR D, G. A., DESENCLOS J C, 2009. International travels and fever screening during epidemics: A literature review on the effectiveness and potential use of non-contact infrared thermometers. Eurosurveillance, 14.

CHO, K. S. & YOON, J. 2014. Fever Screening and Detection of Febrile Arrivals at an International Airport in Korea: Association among Self-reported Fever, Infrared Thermal Camera Scanning, and Tympanic Temperature. Epidemiol Health, 36, e2014004.

GABER, W., GOETSCH, U., DIEL, R., DOERR, H. W. & GOTTSCHALK, R. 2009. Screening for infectious diseases at international airports: the Frankfurt model. Aviat Space Environ Med, 80, 595–600.

GUANGHAO SUN, N. Q. V., SHIGETO ABE, OSAMU TAKEI, MASAMI SUGAMATA, TAKEMI MATSUI, 2013. A Portable Infection Screening System Designed for Onboard Entry Screening Based on Multi-Parameter Vital Signs. International Journal of E-Health and Medical Communications, 3, 20–30.

HUIZER, Y.L., SWAAM, C.M., LEITMEYER, K.C. & TIMEN, A. 2014. Usefulness and applicability of infectious disease control measures in air travel: A review. Travel Med Infect Dis, 13(1), 19–30

JEFFERSON, T., DEL MAR, C., DOOLEY, L., FERRONI, E., AL-ANSARY, L. A., BAWAZEER, G. A., VAN DRIEL, M. L., FOXLEE, R. & RIVETTI, A. 2009. Physical interventions to interrupt or reduce the spread of respiratory viruses: systematic review. BMJ, 339, b3675.

JONES, J., GASTELLU-ETCHEGORRY, M., STENZ, F. K., BAUDON, C., BLOEM, S. J., BONDONNEAU, M., COHUET, S., DIGGLE, R., EWING, R. W., GERSTENBLUTH, I., GRANGEON, J. P., KUMAR ALLA, K., LAJOINIE, G., TROMP, M., TUMAHAI, T., YVON, J. F., SWAAN, C. M. & GOSSNER, C. M. 2011. Epidemiology, surveillance and control of infectious diseases in the European overseas countries and territories, 2011. Euro Surveill, 16.

KRISHNAMURTHY, R., REMIS, M., BROOKE, L., MILLER, C., NAVIN, A. & GUERRA, M. 2006. Quarantine Activity Reporting System (QARS). AMIA Annu Symp Proc, 990.

MCBRIDE, W. J., BUIKSTRA, E. & FITZGERALD, M. 2010. Investigation of febrile passengers detected by infrared thermal scanning at an international airport. Aust N Z J Public Health, 34, 5–10.

MERRILL, R. D., ROGERS, K., WARD, S., OJO, O., KAKAI, C. G., AGBEKO, T. T., GARBA, H., MACGURN, A., OPPERT, M., KONE, I., BAMSA, O., SCHNEIDER, D. & BROWN, C. 2017. Responding to Communicable Diseases in Internationally Mobile Populations at Points of Entry and along Porous Borders, Nigeria, Benin, and Togo. Emerg Infect Dis, 23.

SELVEY, L. A., ANTAO, C. & HALL, R. 2015. Entry screening for infectious diseases in humans. Emerg Infect Dis, 21, 197–201.

SUN, G., ABE, N., SUGIYAMA, Y., NGUYEN, Q. V., NOZAKI, K., NAKAYAMA, Y., TAKEI, O., HAKOZAKI, Y., ABE, S. & MATSUI, T. 2013. Development of an infection screening system for entry inspection at airport quarantine stations using ear temperature, heart and respiration rates. Conf Proc IEEE Eng Med Biol Soc, 2013, 6716–6719.

**Influenza**

CHEN, J., YANG, K., ZHANG, M., SHEN, C., CHEN, J., WANG, G., HUANG, S., ZHANG, M., XIONG, H., CHEN, H., CHEN, Y. & XIA, N. 2018. Rapid identification of imported influenza viruses at Xiamen International Airport via an active surveillance program. Clin Microbiol Infect, 24, 289–294.

COWLING, B. J., LAU, L. L., WU, P., WONG, H. W., FANG, V. J., RILEY, S. & NISHIURA, H. 2010. Entry screening to delay local transmission of 2009 pandemic influenza A (H1N1). BMC Infect Dis, 10, 82.

FUJITA, M., SATO, H., KAKU, K., TOKUNO, S., KANATANI, Y., SUZUKI, S. & SHINOMIYA, N. 2011. Airport quarantine inspection, follow-up observation, and the prevention of pandemic influenza. Aviat Space Environ Med, 82, 782–789.

GOMEZ, J., MUNAYCO, C. V., ARRASCO, J. C., SUAREZ, L., LAGUNA-TORRES, V. A., AGUILAR, P. V., CHOWELL, G. & KOCHEL, T. J. 2009. Pandemic Influenza in a Southern Hemisphere Setting: The Experience in Peru from May to September, 2009. Eurosurveillance, 14, 22–27.

GUNARATNAM, P. J., TOBIN, S., SEALE, H., MARICH, A. & MCANULTY, J. 2014. Airport arrivals screening during pandemic (H1N1) 2009 influenza in New South Wales, Australia. Med J Aust, 200, 290–292.

HALE, M. J., HOSKINS, R. S. & BAKER, M. G. 2012. Screening for influenza A(H1N1)pdm09, Auckland International Airport, New Zealand. Emerg Infect Dis, 18, 866–868.

JIUN-SHIAN KUO, Y.-H. L., JUI-WEI HSIEH, MIN-CHENG LIN, SHIH-YAN YANG, 2009. Initial Evaluation on Screening of Novel Influenza A (H1N1) at International Ports in Taiwan. Taiwan Epidemiology Bulletin, 25, 626–647.

KHAN, K., ECKHARDT, R., BROWNSTEIN, J. S., NAQVI, R., HU, W., KOSSOWSKY, D., SCALES, D., ARINO, J., MACDONALD, M., WANG, J., SEARS, J. & CETRON, M. S. 2013. Entry and exit screening of airline travelers during the A(H1N1) 2009 pandemic: a retrospective evaluation. Bull World Health Organ, 91, 368–376.

LYTRAS, T., THEOCHAROPOULOS, G., TSIODRAS, S., MENTIS, A., PANAGIOTOPOULOS, T., BONOVAS, S. & INFLUENZA SURVEILLANCE REPORT, G. 2009. Enhanced surveillance of influenza A(H1N1)v in Greece during the containment phase. Euro Surveill, 14.

MUKHERJEE, P., LIM, P. L., CHOW, A., BARKHAM, T., SEOW, E., WIN, M. K., CHUA, A., LEO, Y. S. & CHENG CHEN, M. I. 2010. Epidemiology of travel-associated pandemic (H1N1) 2009 infection in 116 patients, Singapore. Emerg Infect Dis, 16, 21–26.

NISHIURA, H. & KAMIYA, K. 2011. Fever screening during the influenza (H1N1-2009) pandemic at Narita International Airport, Japan. BMC Infect Dis, 11, 111.

NISHIURA, H., WILSON, N. & BAKER, M. G. 2009. Quarantine for pandemic influenza control at the borders of small island nations. BMC Infectious Diseases, 9.

PRIEST, P. C., DUNCAN, A. R., JENNINGS, L. C. & BAKER, M. G. 2011. Thermal image scanning for influenza border screening: results of an airport screening study. PLoS One, 6, e14490.

PRIEST, P. C., JENNINGS, L. C., DUNCAN, A. R., BRUNTON, C. R. & BAKER, M. G. 2013. Effectiveness of border screening for detecting influenza in arriving airline travelers. Am J Public Health, 103, 1412–1418.

SAKAGUCHI, H., TSUNODA, M., WADA, K., OHTA, H., KAWASHIMA, M., YOSHINO, Y. & AIZAWA, Y. 2012. Assessment of Border Control Measures and Community Containment Measures Used in Japan during the Early Stages of Pandemic (H1N1) 2009. Plos One, 7.

SCHLAICH, C., SEVENICH, C. & GAU, B. 2012. [Public health measures at the airport of Hamburg during the early phase of pandemic influenza (H1N1) 2009]. Gesundheitswesen, 74, 145–153.

SHIMADA, T., GU, Y., KAMIYA, H., KOMIYA, N., ODAIRA, F., SUNAGAWA, T., TAKAHASHI, H., TOYOKAWA, T., TSUCHIHASHI, Y., YASUI, Y., TADA, Y. & OKABE, N. 2009. Epidemiology of influenza A(H1N1)v virus infection in Japan, May-June 2009. Euro Surveill, 14.

WORLD HEALTH ORGANISATION 2009a. Responding to New Influenza A(H1N1): Options for interventions at international points of entry (interim option paper). In: PACIFIC, W. R. O. F. T. W. (ed.).

WORLD HEALTH ORGANISATION 2009b. WHO technical advice for case management of Influenza A(H1N1) in air transport, Developed in cooperation with The International Civil Aviation Organisation and The International Air Transport Association.

WORLD HEALTH ORGANISATION 2010b. Public health measures taken at international borders during early stages of pandemic influenza A (H1N1) 2009: preliminary results. Weekly epidemiological record, 21.

ZHANG, Y., YANG, P., LIYANAGE, S., SEALE, H., DENG, Y., PANG, X., TIAN, L., LIU, B., ZHANG, L. & WANG, Q. 2012. The characteristics of imported cases and the effectiveness of outbreak control strategies of pandemic influenza A (H1N1) in China. Asia Pac J Public Health, 24, 932–939.

**Pandemic influenza preparedness**

BELL, D., NICOLL, A., FUKUDA, K., HORBY, P., MONTO, A. & GRP, W. H. O. W. 2006. Nonpharmaceutical interventions for pandemic influenza, international measures. Emerging Infectious Diseases, 12, 81–87.

FEDERAL REPUBLIC OF NIGERIA 2013. Nigeria National Pandemic Influenza Preparedness and Response Plan.

HOMELAND SECURITY COUNCIL USA 2006. National strategy for pandemic influenza Implementation plan.

MALONE, J. D., BRIGANTIC, R., MULLER, G. A., GADGIL, A., DELP, W., MCMAHON, B. H., LEE, R., KULESZ, J. & MIHELIC, F. M. 2009. US airport entry screening in response to pandemic influenza: modeling and analysis. Travel Med Infect Dis, 7, 181–191.

MCLEOD, M., KELLY, H., WILSON, N. & BAKER, M. G. 2008. Border control measures in the influenza pandemic plans of six South Pacific nations: a critical review. N Z Med J, 121, 62–72.

MOUNIER-JACK, S., JAS, R. & COKER, R. 2007. Progress and shortcomings in European national strategic plans for pandemic influenza. Bull World Health Organ, 85, 923–929.

WORLD HEALTH ORGANISATION 2005. WHO global influenza preparedness plan. The role of WHO and recommendations for national measures before and during pandemics.

WORLD HEALTH ORGANISATION 2017b. Pandemic Influenza Risk Management: A WHO guide to inform and harmonize national and international pandemic preparedness and response, Geneva: World Health Organisation.

WORLD HEALTH ORGANISATION 2018a. A checklist for pandemic influenza risk and impact management: building capacity for pandemic response, Geneva, World Health Organisation.

**International air travel and infectious diseases**

BRANNEN, D. E., ALHAMMAD, A., BRANUM, M. & SCHMITT, A. 2016. International Air Travel to Ohio, USA, and the Impact on Malaria, Influenza, and Hepatitis A. Scientifica (Cairo), 2016, 8258946.

**Preparedness planning for infectious disease**

BERNITZ, B. K. 2008. Communicable disease policy development in response to changing European political frontiers in Finland, Norway, and Sweden. Scand J Public Health, 36, 875–878.

CHIU, H. H., HSIEH, J. W., WU, Y. C., CHOU, J. H. & CHANG, F. Y. 2014. Maintaining Human Health at the Border of Taiwan. Biosecurity and Bioterrorism-Biodefense Strategy Practice and Science, 12, 346–355.

WARREN, A., BELL, M. & BUDD, L. 2012. Model of health? Distributed preparedness and multi-agency interventions surrounding UK regional airports. Soc Sci Med, 74, 220–227.

WORLD HEALTH ORGANISATION 2010a. Protocol for Assessing National Surveillance and Response Capacities for the International Health Regulations (2005).

WORLD HEALTH ORGANISATION 2011. International Health Regulations (2005), IHR Core capacity monitoring framework: Checklist and Indicators for Monitoring Progress in the Development of IHR Core Capacities in States Parties.

WORLD HEALTH ORGANISATION 2012. International health regulations (2005): a guide for public health emergency contingency planning at designated points of entry.

WORLD HEALTH ORGANISATION 2013a. Activity Report, Review in activities 2012, Global Outbreak and Alert Response, World Health Organisation.

WORLD HEALTH ORGANISATION 2013b. A Systematic Review of Public Health Emergency Operations Centers (EOC).

WORLD HEALTH ORGANISATION 2016. Handbook for management of public health events on board ships.

WORLD HEALTH ORGANISATION 2017a. Joint External Evaluation of IHR Core Capacities of the Federal Republic of Nigeria, Geneva: World Health Organisation.

WORLD HEALTH ORGANISATION 2018b. Coordination of public health surveillance between points of entry and the national public health surveillance system Advising principles.

WORLD HEALTH ORGANISATION 2018c. Joint external evaluation tool: International Health Regulations (2005), second edition, Geneva: World Health Organisation.

WORLD HEALTH ORGANISATION 2009c. International Health Regulations (2005), Assessment tool for core capacity requirements at designated airports, ports, and ground crossings.

**Screening measures at ground crossing**

CLEARY, V., WYNNE-EVANS, E., FREED, J., FLEET, K., THORN, S. & TURBITT, D. 2017. Ebola 2014: Setting up a port health screening programme at an international train station. J Bus Contin Emer Plan, 10, 177–187.

**Sever Acute Respiratory Syndrome**

BELL, D. M., WORLD HEALTH ORGANISATION WORKING GROUP ON, I. & COMMUNITY TRANSMISSION OF, S. 2004. Public health interventions and SARS spread, 2003. Emerg Infect Dis, 10, 1900–1906.

GLASS, K. & BECKER, N. G. 2006. Evaluation of measures to reduce international spread of SARS. Epidemiol Infect, 134, 1092–1101.

LEE, C. W., TSAI, Y. S., WONG, T. W. & LAU, C. C. 2006. A loophole in international quarantine procedures disclosed during the SARS crisis. Travel Med Infect Dis, 4, 22–28.

PITMAN, R. J., COOPER, B. S., TROTTER, C. L., GAY, N. J. & EDMUNDS, W. J. 2005. Entry screening for severe acute respiratory syndrome (SARS) or influenza: policy evaluation. BMJ, 331, 1242–1243.

SAMAAN, G., PATEL, M., SPENCER, J. & ROBERTS, L. 2004. Border screening for SARS in Australia: what has been learnt? Med J Aust, 180, 220–223.

ST JOHN, R. K., KING, A., DE JONG, D., BODIE-COLLINS, M., SQUIRES, S. G. & TAM, T. W. 2005. Border screening for SARS. Emerg Infect Dis, 11, 6–10.

TAN, C. C. 2006. SARS in Singapore—Key lessons from an epidemic. Annals Academy of Medicine Singapore, 35, 345–349.

VENKATESH, S. & MEMISH, Z. A. 2004. SARS: the new challenge to international health and travel medicine. East Mediterr Health J, 10, 655–662.

WILDER-SMITH, A., GOH, K. T. & PATON, N. I. 2003. Experience of severe acute respiratory syndrome in Singapore: Importation of cases, and defense strategies at the airport. Journal of Travel Medicine, 10, 259–262.

WORLD HEALTH ORGANISATION 2003. WHO issues consensus document on the epidemiology of SARS. Wkly Epidemiol Rec, 78, 373–375.

**Zika Virus Disease**

HO, L. L., TSAI, Y. H., LEE, W. P., LIAO, S. T., WU, L. G. & WU, Y. C. 2017. Taiwan’s Travel and Border Health Measures in Response to Zika. Health Secur, 15, 185–191.

HUANG, A. S. E., SHU, P. Y. & YANG, C. H. 2016. A new reportable disease is born: Taiwan Centers for Disease Control’s response to emerging Zika virus infection. Journal of the Formosan Medical Association, 115, 223–225.

## Appendix F

**Table A2:** WHO Temporary recommendations for PHEIC and advice for response measures to other PHE.

Public Health Event		WHO Statement/Date
Title	Started/Ended	
Ebola Virus Disease outbreak in West Africa	8 Aug 2014/29 Mar 2016	**Statement on the 1st/**8 Aug 2014 **Statement on the 2nd meeting/**22 September 2014 **Statement on the 3rd meeting/**23 October 2014 **Statement on the 4th meeting/**21 January 2015 **Statement on the 5th meeting/**10 April 2015 **Statement on the 6th meeting/**7 July 2015 **Statement on the 7th meeting/**5 October 2015 **Statement on the 8th meeting/**18 December 2015 **Statement on the 9th meeting/**29 March 2016
Poliovirus	5 May 2014/15 Aug 2018	**Statement on the 3rd meeting/**14 November 2014 **Statement on the 6th meeting/**17 August 2015 **Statement on the 7th meeting/**26 November 2015 **Statement on the 8th meeting/**1 March 2016 **Statement on the 9th meeting/**20 May 2016 **Statement on the 10th meeting/**22 August 2016 **Statement on the 11th meeting/**11 November 2016 **Statement on the 12th meeting/**13 February 2017 **Statement on the 13th meeting/**2 May 2017 **Statement on the 14th meeting/**3 August 2017 **Statement on the 15th meeting/**14 November 2017 **Statement on the 16th meeting/**14 February 2018 **Statement on the 17th meeting/**10 May 2018 **Statement on the 18th meeting/**15 Aug 2018 **Statement on the 19th meeting/**30 Nov 2018
Ebola outbreak in DRC	10 May 2018/25 July 2018	**Statement on the 1st meeting/**18 May 2018 **External situation report/**11,14,18,20,25,29 May 2018 **External situation report/**1,5,8,12,19,22,26 June 2018 **External situation report/**1,12,25 July 2018
	4 Aug 2018/5 Dec 2018, ongoing	**External situation report/**7,14,22,28 Aug 2018 **External situation report/**4,11,18,25 Sept 2018 **External situation report/**4,9,17,23,30 Oct 2018 **External situation report/**6,13,21,28 Nov 2018 **External situation report/**5 Dec 2018
MERS-CoV	9 July 2013/3 Sept 2015	**WHO statement on the 10th meeting of the IHR Emergency Committee regarding MERS/** 3 September 2015
Yellow fever	31 Aug 2016/16 May 2017	-
Zika virus	8 Mar 2016/18 Nov 2016	**Statement on the 1st meeting/**18 November 2016 **WHO statement on the 2nd meeting/**2 September 2016 **WHO statement on the 3rd meeting/**14 June 2016 **WHO statement on the 4th meeting/**8 March 2016 **WHO statement on the 5th meeting/**1 February 2016
Plague	4 Oct 2017/4 Dec 2017	**External situation report/**4 October 2017 **External situation report/**9, 12, 17, 20, 26, 31 October 2017 **External situation report/**6, 9, 14, 17, 20, 27 November 2017 **External situation report/**4 December 2017
SARS	27 Mar 2003/24 Jun 2003	**Update 11—WHO recommends new measures to prevent travel-related spread of SARS/** 27 March 2003 **Update 37—WHO extends its SARS-related travel advice to Beijing and Shanxi Province in China and to Toronto Canada/**23 April 2003 **Update 42—Travel advice for Toronto, situation in China/**29 April 2003 **Update 50—WHO extends its SARS-related travel advice to Tianjin, Inner Mongolia and Taipei in China/**8 May 2003 **Update 58—First global consultation on SARS epidemiology, travel recommendations for Hebei Province (China), situation in Singapore/**17 May 2003 **SARS Travel Recommendations/**10 June 2003 **Update 80—Change in travel recommendations for parts of China, situation in Toronto/**13 June 2003 **SARS Travel Recommendations/**16 June 2003 **Update 82—Change in travel recommendations for Taiwan/**17 June 2003 **Update 86—Hong Kong removed from list of areas with local transmission/**23 June 2003 **Update 87—World Health Organisation changes last remaining travel recommendation—for Beijing, China/**24 June 2003
Influenza Pandemic (H1N1) 2009	25 Apr 2009/10 Aug 2010	**1st meeting/**25 April 2009 **2nd meeting/**27 April 2009 **3rd meeting/**5 June 2009 **4th meeting/**11 June 2009 **5th meeting/**24 September 2009 **6th meeting/**26 November 2009 **7th meeting/**24 February 2010 **8th meeting/**3 June 2010 **9th meeting/**10 August 2010
